# Triplex DNA-binding proteins are associated with clinical outcomes revealed by proteomic measurements in patients with colorectal cancer

**DOI:** 10.1186/1476-4598-11-38

**Published:** 2012-06-08

**Authors:** Laura D Nelson, Christian Bender, Heiko Mannsperger, Daniel Buergy, Patryk Kambakamba, Giridhar Mudduluru, Ulrike Korf, Dennis Hughes, Michael W Van Dyke, Heike Allgayer

**Affiliations:** 1 Dept. of Pediatrics, The University of Texas M.D. Anderson Cancer Center, Houston, TX 77030, USA; 2 Molecular Genome Analysis, German Cancer Research Center, Heidelberg, Germany; 3 Dept. of Anesthesiology and Intensive Care Medicine, Medical Faculty Mannheim, University of Heidelberg, Heidelberg, Germany; 4 Experimental Surgery, Medical Faculty Mannheim, University of Heidelberg, Mannheim, Germany; 5 Dept of Chemistry and Physics, Western Carolina University, Cullowhee, NC 28723, USA; 6 Molecular Oncology of Solid Tumors, German Cancer Research Center, Heidelberg, Germany

## Abstract

**Background:**

Tri- and tetra-nucleotide repeats in mammalian genomes can induce formation of alternative non-B DNA structures such as triplexes and guanine (G)-quadruplexes. These structures can induce mutagenesis, chromosomal translocations and genomic instability. We wanted to determine if proteins that bind triplex DNA structures are quantitatively or qualitatively different between colorectal tumor and adjacent normal tissue and if this binding activity correlates with patient clinical characteristics.

**Methods:**

Extracts from 63 human colorectal tumor and adjacent normal tissues were examined by gel shifts (EMSA) for triplex DNA-binding proteins, which were correlated with clinicopathological tumor characteristics using the Mann-Whitney *U*, Spearman’s rho, Kaplan-Meier and Mantel-Cox log-rank tests. Biotinylated triplex DNA and streptavidin agarose affinity binding were used to purify triplex-binding proteins in RKO cells. Western blotting and reverse-phase protein array were used to measure protein expression in tissue extracts.

**Results:**

Increased triplex DNA-binding activity in tumor extracts correlated significantly with lymphatic disease, metastasis, and reduced overall survival. We identified three multifunctional splicing factors with biotinylated triplex DNA affinity: U2AF65 in cytoplasmic extracts, and PSF and p54nrb in nuclear extracts. Super-shift EMSA with anti-U2AF65 antibodies produced a shifted band of the major EMSA H3 complex, identifying U2AF65 as the protein present in the major EMSA band. U2AF65 expression correlated significantly with EMSA H3 values in all extracts and was higher in extracts from Stage III/IV vs. Stage I/II colon tumors (p = 0.024). EMSA H3 values and U2AF65 expression also correlated significantly with GSK3 beta, beta-catenin, and NF- B p65 expression, whereas p54nrb and PSF expression correlated with c-Myc, cyclin D1, and CDK4. EMSA values and expression of all three splicing factors correlated with ErbB1, mTOR, PTEN, and Stat5. Western blots confirmed that full-length and truncated beta-catenin expression correlated with U2AF65 expression in tumor extracts.

**Conclusions:**

Increased triplex DNA-binding activity *in vitro* correlates with lymph node disease, metastasis, and reduced overall survival in colorectal cancer, and increased U2AF65 expression is associated with total and truncated beta-catenin expression in high-stage colorectal tumors.

## Background

DNA and RNA are dynamic molecules that adopt several different secondary and tertiary structures. DNA can form a stable triple helix in which a purine- or pyrimidine-rich third strand forms sequence-specific H-bonds (Hoogsteen and reverse-Hoogsteen) with a purine-rich strand in the major groove of the Watson-Crick duplex in polypyrimidine-polypurine repeat sequences [1]. Guanine (G)-rich DNA and RNA can also form G-quadruplexes that also use Hoogsteen and reverse Hoogsteen G*G bonds in a non-canonical four-stranded topology. G-quadruplexes specifically have been implicated at DNA telomere ends, the purine-rich DNA strands of oncogenic promoters, and in RNA 5’-untranslated regions (UTR) near translation start sites [2]. For example, a nuclease-sensitive element in the human *c-MYC* promoter that can form either a DNA triplex or G-quadruplex interferes with DNA transcription [3]. Transient Hoogsteen base pairs have been detected in DNA duplexes bound to transcription factors and in damaged DNA, suggesting that the DNA double helix can resonate and form excited-state Hoogsteen base pairs that can expand its structural complexity [4].

Genomic instability in association with carcinogenesis is well established and promotes multiple hallmarks of cancer [5]. Repetitive DNA, such as tri- and tetranucleotide sequences, is genetically unstable, and expansions of such DNA repeats are associated with numerous hereditary neurological diseases including Fragile X syndrome, myotonic dystrophy, and Friedreich’s ataxia [6,7]. Many of these DNA repeat sequences can exist in at least two different conformations, and at least 10 non-B DNA conformations can form, perhaps transiently, at specific sequences due to negative supercoiling generated by DNA replication, transcription, protein binding, or during DNA repair [8]. Non-B DNA structures such as cruciforms, triplexes and G-quadruplexes can cause mutations such as deletions, expansions, and translocations [9,10]. Bacolla *et al.* found that genes containing long polypyrimidine-polypurine sequences are more susceptible to chromosomal translocations than genes that do not contain these sequences [11]. Researchers have located “hotspot” regions of the genome at or near sequences with the potential to form non-B DNA structures, including the region in the promoter of the human *c-MYC* gene capable of forming triplex or G-quadruplex DNA that overlaps with one of the major breakpoint hotspots in *c-MYC*-induced lymphomas and leukemias [12,13]. The recently created Non-B Database ( http://nonb.abcc.ncifcrf.gov) can be used to predict the capability of a DNA sequence in mammalian genomes to form any of a variety of non-B structures [14].

While the existence of triplex or G-quadruplex nucleic acids *in vivo* has yet to achieve mainstream acceptance, eukaryotic proteins that recognize and bind to these alternative structures do exist. For example, the Fragile X mental retardation protein (FMRP) binds an intramolecular G-quartet in target mRNAs, and loss of function of this protein causes the Fragile X mental retardation syndrome [15]. We have studied proteins in *Saccharomyces cerevisiae* and HeLa carcinoma cells that bind specifically to a purine-motif triplex DNA probe in gel shifts (EMSA) where the third strand is G-rich and photo-crosslinked with a psoralen group (Ps~) [16-18]. Stm1, the major purine-motif triplex DNA-binding protein in *S. cerevisiae*, also binds to G-quartet DNA and RNA *in vitro*[19]. Using Southwestern blotting where HeLa nuclear extracts were separated by SDS-PAGE, blotted and probed with the same radio-labeled purine triplex DNA used in EMSA, we found that 100-, 60-, and 15-kDa bands were hybridized with the triplex DNA probe, whereas only the 100-kDa band was also hybridized with the parent duplex DNA probe [16]. RecQ-family helicases, including the WRN helicase, have been shown to preferentially bind to and unwind aberrant DNA structures such as triplex and G-quadruplex DNAs, which are believed to exist *in vivo* as intermediates in DNA replication, recombination, and repair. The WRN helicase is deficient in patients with Werner syndrome, an autosomal recessive disease causing premature aging that is associated with numerous age-related phenotypes, including a high predisposition to cancer [20]. Others have examined specific aspects of WRN expression in colorectal cancer, such as the presence of allelic variants and colorectal cancer risk and WRN promoter methylation as it correlates with a CpG island methylation phenotype (CIMP)-high diagnosis [21,22]. These studies led us to question whether triplex DNA-binding proteins and WRN helicase expression are quantitatively and/or qualitatively different in human colorectal tumors and corresponding normal tissues, if there is any correlation with clinical prognosis, and identify purine-motif triplex DNA-binding proteins in human cells.

Numerous genetic, cytogenetic, and epigenetic aberrations act at specific stages in colorectal cancer initiation and progression and influence response to therapy, such as inactivation of tumor suppressor APC as an initiating event and *KRAS* or *BRAF* mutations as markers of non-response to EGFR-targeted therapy [23]. High-throughput studies have suggested the existence of additional undiscovered cancer genes that may promote colorectal cancer development [24-26]. Colorectal cancer is also one of the more genetically unstable cancers, with about 65% of sporadic adenomas and cancers being characterized by chromosomal instability (CIN), 10-15% characterized by microsatellite instability (MSI), and approximately 20% having a CIMP phenotype, with some overlap among these characteristics.

We have found higher triplex DNA-binding activity *in vitro* in colorectal tumor extracts than in corresponding normal tissue extracts using EMSA, and that this increased binding activity correlated significantly with the spread of cancer to the lymph nodes, metastasis, and reduced overall survival. We also found that expression of the triplex/G-quadruplex-unwinding helicase WRN correlated significantly with total triplex DNA-binding activity in EMSAs in both normal and tumor tissue extracts. Biotin purine-motif triplex DNA affinity identified three multifunctional splicing factors: U2AF65, PSF, and p54nrb, and an anti-U2AF65 antibody produced a super-shifted EMSA band. High U2AF65 expression was associated with advanced colon tumor stages and with p54nrb and PSF expression in tumors. U2AF65 expression also correlated significantly with both total and truncated beta-catenin, as well as NF- B p65, PCNA, EGFR, mTOR, PTEN, and Stat5 in colorectal tumors.

## Materials and methods

Preparation of cytoplasmic and nuclear extracts of tissue and cell lines. Tissue samples of tumor and adjacent normal mucosa were collected after surgical resections after informed consent, verification by a pathologist, and snap-frozen in liquid nitrogen. The patients had not previously received any chemotherapy, therefore the tissues are chemotherapy naïve. Frozen tissue samples were prepared as described by Asangani et al. [27]. The samples were pulverized with a Sartorius Mikrodismembrator, then extracted for 30 min on ice with Schaffner lysis buffer A (10 mM HEPES-Na + pH 7.9, 10 mM KCl, 0.1 mM EDTA pH 8.0, 0.1 mM EGTA pH 8, 1 mM dithiothreitol, 0.5% Triton X-100, Sigma phosphatase inhibitor cocktail 2, and Roche Complete Mini protease inhibitor) and centrifuged at 13,000 rpm, 4°C in a microcentrifuge to produce cytoplasmic extracts. The nuclear pellet was extracted for 30 min on ice with Schaffner buffer C (20 mM HEPES-Na + pH 7.9, 0.4 M NaCl, 0.1 mM EDTA pH 8.0, 0.1 mM EGTA pH 8.0, 1 mM dithiothreitol, 20% glycerol, with phosphatase and protease inhibitors) and centrifuged at 13,000 rpm, 4°C in a microcentrifuge to produce nuclear extracts [28]. Total protein concentrations were determined using the Pierce BCA Protein Assay kit. Colorectal cancer cell lines and HeLa cytoplasmic or nuclear extracts were similarly prepared using Schaffner buffers A and C, respectively.

### Purine-motif triplex DNA formation and ^33^P-labeling

Purine triplex DNA oligonucleotide sequences and probe formation were as previously described [16,17]. The parent duplex oligonucleotides are PuGA: 5’ – AATTCCTAAGGGAGGGGAGGGGAGGGTAGCT – 3’ and complementary strand PuCT: 5’ – AGCTACCCTCCCCTCCCCTCCCTTAGG – 3’. The parent duplex DNA was made by annealing equimolar (0.1 mM) concentrations of the PuGA and PuCT oligonucleotides at room temperature after boiling for 2 min in 40 mM Tris-HCl pH 8.0, 10 mM MgCl_2_, 0.01% NP-40. The purine-motif triplex-forming oligonucleotide (TFO) contained a 4’-(hydroxymethyl)-4,5’,8-trimethylpsoralen-hexyl (Ps~) moiety at the 5’-terminus (Eurogentec): 5’ – Ps ~ GGG TGG GGT GGG GTG GGT -3’. To form triplex DNA, the parent duplex DNA and a 10-fold molar excess of TFO were incubated for 4 h at 30°C in 40 mM Tris HCl pH 8.0, 100 mM MgCl_2_, 0.01% NP-40. Psoralenated TFO was then cross-inked with the parent DNA duplex with a 366 nm UV transilluminator for 10 min on ice. Purine triplex DNA (1 x 10^-7^ M) was 3’ end-labeled with T4 kinase (New England Biolabs) and γ-^33^P dATP for 1 h at 37°C. Unincorporated labeling dATP was removed from the reaction by centrifuging the reaction mixture with an equal volume of 10 mM Tris-HCl pH 8.0, 10 mM MgCl_2_, 0.05% Triton X-100 through a G25 Microspin column (GE Healthcare).

### Electrophoretic mobility shift assay (EMSA) and super-shift EMSA

Gel shifts were also done as previously described [16,17]. In this study 5 μg total protein from tissue extracts or 1.5 μg HeLa or colorectal cancer cell line cytoplasmic or nuclear extracts were mixed with 1 nM ^33^P-labeled purine triplex DNA and 2 μg poly (dIdC) carrier DNA in binding buffer (25 mM HEPES-Na + pH 7.9, 50 mM KCl, 10% glycerol, 0.5 mM dithiothreitol, 2 mM MgCl_2_) for 30 min at room temperature. Protein-triplex DNA probe complexes were resolved by nondenaturing PAGE at 7 V/cm for 90 min through a 5% acrylamide/0.25% bisacrylamide gel containing 22 mM Tris borate, 0.5 mM EDTA, and 5% glycerol. Protein-probe complexes were visualized using autoradiography and quantitated with a Storm 840 PhosphorImager (Molecular Dynamics). Major EMSA H3 bands from each tissue sample were normalized by dividing by the H3 band value of HeLa nuclear extract present in each gel. For super-shift EMSA, protein extracts were incubated in the same binding buffer with purine triplex DNA probe for 30 min at room temperature, then 400 ng of anti-U2AF65 MC3 antibody or mouse IgG antibody as a negative control (Santa Cruz) were added to the reaction and incubated for 1 h at room temperature. PAGE gels were run as for regular EMSA with the addition of a circulating cooling water bath to the gel apparatus.

### Statistical correlations

The Wilcoxon Sign Rank Test was used to compare the level of the major EMSA H3 complex and WRN expression in total, cytoplasmic, and nuclear extracts of colorectal tumors and corresponding normal tissues. The Mann-Whitney *U* test was used with SPSS version 13.0 to compare quantitative variables in two independent groups. Spearman correlations among continuous variables were computed. Chi square (Bonferroni-corrected) were used for grouped/dichotomized variables. Survival was estimated using Kaplan-Meier analysis, and differences were calculated using Mantel-Cox log-rank statistics; primary endpoints were tumor-related death (disease-specific survival), death (overall survival), and tumor recurrence (recurrence-free survival, R0-patients only). The following variables were dichotomized according to the median value: protein levels in nuclear and total extracts (cytoplasm and nucleus) ratios (tumor/normal) as high levels in tumor (values above the median) vs. low levels in tumor (values below the median) as compared with normal tissue, involved lymph nodes as pN0 vs. pN1-3, distant metastasis as M0 vs. M1, surgical curability as curative vs. non-curative resection (R0 vs. R1/2).

### Purification of triplex DNA-binding proteins using biotin/streptavidin affinity

Biotinylated purine triplex DNA was formed using a 3’ biotinylated PuCT oligonucleotide (Eurogentec): 5’ – AGCTACCCTCCCCTCCCCTCCCTTAGGAATTTT-biotin-3’ annealed to the PuGA complementary strand, then annealed and crosslinked with the Ps ~ TFO as described above. Purification of DNA-binding proteins using biotin/streptavidin affinity systems, as described in Current Protocols in Molecular Biology [29], was performed in separate 2 ml reactions containing either 800 μg RKO colorectal cancer cell nuclear extract or 1085 μg RKO cytoplasmic extract, EMSA binding buffer (25 mM HEPES-Na + pH 7.9, 50 mM KCl, 10% glycerol, 0.5 mM dithiothreitol, 2 mM MgCl_2_), 600 μg poly (dIdC), 1 nM biotinylated purine triplex DNA, and 150 μl pretreated streptavidin agarose (Fluka) while rotating for 2 hr at room temperature. Streptavidin agarose was gently pelleted and washed three times with binding buffer. Laemmli buffer was added directly to the agarose pellet and boiled for 5 min to elute bound protein(s). Proteins were separated using 10% SDS-PAGE and stained with Coomassie blue. Two bands (100 and 60 kDa) from the nuclear extract reaction and one band (65 kDa) from the cytoplasmic extract reaction were excised from the gel and submitted to the German Cancer Research Center (DKFZ) Functional Proteome Analysis laboratory for sequencing and analysis using nano-HPLC ESI-MS-MS and identified using MASCOT database searches.

### Western blotting

Western blot analysis was performed using standard procedures as described in Current Protocols in Molecular Biology [27]. 25 μg total protein from tissue or cell line cytoplasmic or nuclear extract was separated by 10% SDS-PAGE, then electro-transferred to nitrocellulose membranes in 25 mM Tris, 190 mM glycine with 20% methanol. After blocking in 5% milk in Tris-buffered saline with 0.2% Tween-20 (TBST) for 1 hr at room temperature, membranes were incubated with antibodies against WRN (H-300 Santa Cruz sc-5629, 1:500), U2AF65 (MC3 Santa Cruz sc-53942, 1:2000), PSF (39-1 Santa Cruz sc-101137, 1:2000), p54nrb (H-85 Santa Cruz sc-67016, 1:2000) in 5% milk-TBST for 1 hr at room temperature, or beta-catenin (L87A12 Cell Signaling CS-2698, 1:1000) or actin (Sigma A2066, 1:1000) in 5% milk in TBST overnight at 4°C. Blots were washed with TBST, incubated with the appropriate HRP-conjugated secondary antibody at 1:4500, and detected by enhanced chemiluminescence (Pierce, Thermo Scientific) and autoradiography. Protein bands were quantitated by densitometry using NIH Image J software and normalized to actin.

### Reverse phase protein array (RPPA)

RPPA was performed as described by Mannsperger et al. [30]. 2.7 ng cytoplasm or 2.8 ng nuclear protein extract per spot was printed with a non-contact spotter onto nitrocellulose slides (Oncyte Avid, Grace Bio-labs, Bend OR) using an Aushon 2470 Microarrayer (Billerica, MA). Slides were mounted in a customized incubation chamber (Metecon, Mannheim Germany), blocked for 1 hr at room temperature with 50% (v/v) Odyssey blocking buffer in PBS and individually stained with 37 validated primary antibodies at 1:300 in blocking buffer at 4°C overnight and Alexa 680-labeled secondary antibodies (Invitrogen) at 1:8000 in PBS with 0.05% Tween for 1 hr at room temperature. Slides were scanned with the Licor Odyssey system and spot intensities were calculated with GenePix Pro 5.0 microarray analysis software (Molecular Devices). To estimate the total protein concentration per spot, a slide from each run was stained with Fast Green FCF (Sigma-Aldrich) as described by Loebke et al. [31]. Data analysis was done using R with the RPPanalyzer package from CRAN ( http://cran.r-project.org, [32]). For each antibody the logged mean of the raw foreground pixel intensities of a single spot was subtracted by the corresponding logged Fast Green FCF signal to normalize for the total protein per spot.

## Results

### Colorectal tumors have higher triplex DNA-binding activity than corresponding normal tissue

**Figure 1:**
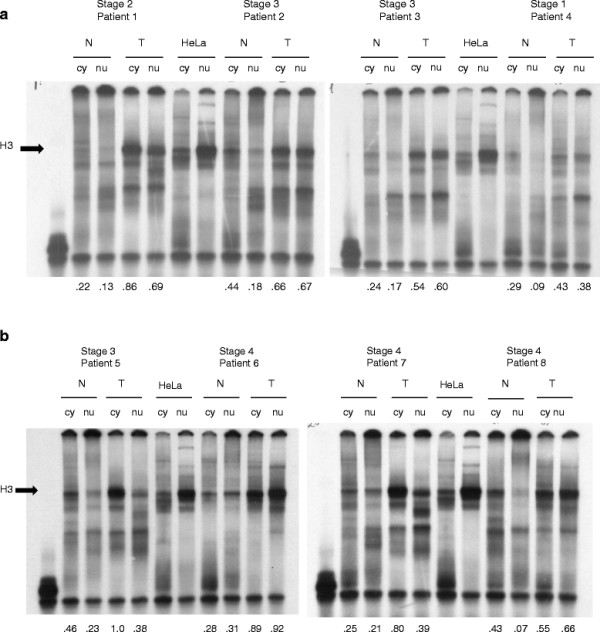
**Electrophoretic Mobility Shift Assay (EMSA) of Resected Tissue Extracts with Purine Triplex DNA. **^33^P-labeled purine-motif triplex DNA (1 nM) was complexed with 5 μg total protein from normal cytoplasmic (N cy), normal nuclear (N nu), tumor cytoplasmic (T cy) or tumor nuclear (T nu) extracts of tissues obtained from eight selected colorectal cancer patients. 1.25 μg HeLa cytoplasmic and nuclear extracts were used as positive (+) controls. The major EMSA band was H3, indicated with an arrow. Normalized EMSA H3 values are listed below the corresponding samples. The purine-motif triplex probe alone is shown in lane 1.

**Figure 2:**
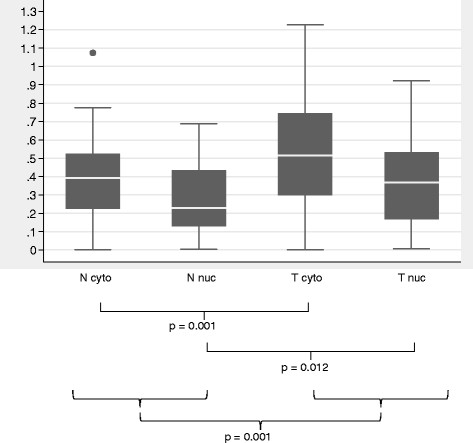
**Normalized EMSA H3 values for all 63 colorectal cancer patient extracts. **Box plots indicating the median normalized EMSA H3 values (white lines in the boxes), upper and lower quartiles (25th through 75^th^ percentiles defined by the shaded box), and ranges of data values for each extract type. N cyto, cytoplasmic normal tissue extracts; N nuc, nuclear normal tissue extracts; T cyto, cytoplasmic tumor tissue extracts; Tnuc, nuclear tumor tissue extracts.

A summary of clinical characteristics of the 63 study patients are shown in Table 1. To examine purine-motif triplex DNA-binding proteins, cytoplasmic and nuclear extracts from 63 colorectal cancer patients’ tumor and corresponding normal tissues were isolated and examined by gel shifts (EMSA). Figure 1 presents examples of EMSAs from eight patients representing all four tumor stages, where in most samples one major band (H3) is present in varying amounts. In some patients, tumor cytoplasmic extracts contained a higher amount of the major H3 complex than normal or tumor nuclear extracts (patients 1 and 5), while in other patients, tumor nuclear extracts contained a higher amount of the major H3 complex (patients 6 and 8). Cytoplasmic and nuclear extracts from HeLa cells were included as positive controls. Normalized EMSA H3 values are listed below each sample. To verify that the major EMSA H3 band is specific for the triplex DNA probe, the ^33^P-labeled parent duplex DNA probe lacking G*G base pairs did not produce the major H3 complex in patient tissue or HeLa nuclear extracts (Additional file 1: Figure S1). EMSA H3 binding values were generally higher in tumor than normal tissue, whether evaluating cytoplasmic extracts (mean = 0.512, median = 0.509 for tumor tissue; mean = 0.386, median = 0.384 for normal tissue) or nuclear extracts (mean = 0.361, median = 0.368 for tumor tissue; mean = 0.264, median = 0.228 for normal tissue) as shown in Figure 2. Wilcoxon sign rank test results showed significantly higher triplex DNA EMSA binding activity in tumor than normal extracts when examining total measures (p = 0.001), cytoplasmic extracts only (p = 0.001) and nuclear extracts only (p = 0.012)(Additional file 2). We also performed EMSA analysis of cytoplasmic and nuclear extracts of eight colorectal cancer cell lines (GEO, SW480, HT29, HCT116, Colo206F, wiDR, Colo320, and RKO) and found that all eight cell lines had a triplex DNA-binding protein pattern that was very similar to HeLa extracts, with a moderate amount of the major H3 band produced by cytoplasmic extracts and an abundant amount of the H3 band produced by nuclear extracts (Additional file 1: Figure S2a).

**Table 1:** Patient clinical characteristics

**Tumor characteristics**		**Absolute (n = 63)**	**Relative (%)**
Sex	Male	46	73%
	Female	17	27%
Localization	Colon	36	57.1%
	Rectum	27	42.9%
TNM Staging	pT1	10	15.9%
	pT2	5	7.9%
	pT3	34	54%
	pT4	16	25.4%
Lymph Node Status	pN0	37	58.7%
	pN1	16	25.4%
	pN2	10	15.9%
Metastasis Staging	M0	42	66.7%
	M1	21	33.3%
Status	alive	43	68.1%
	dead	20	31.7%

### Increased triplex DNA-binding activity in colorectal tumors correlates with lymph node disease, metastasis, and overall survival

**Figure 3:**
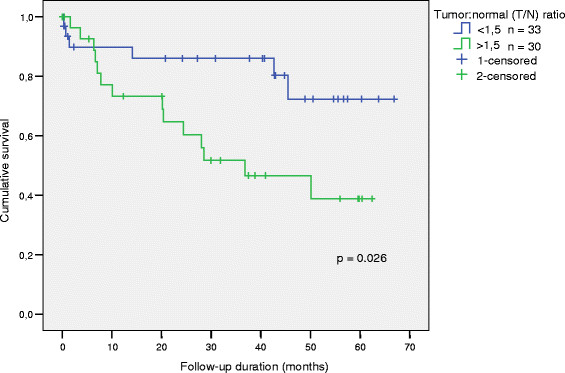
**Overall Survival according to the tumor:normal (T/N) colorectal tissue nuclear triplex DNA-binding activity ratio. **Cut-off = 1.5 (rounded-up median).

We wanted to investigate whether the amount of the EMSA H3 complex correlated with patient clinicopathological data and overall survival. Median follow-up time for patient clinical data was 28.9 months. Normalized EMSA data of patient samples were correlated with clinical risk factors and computed for univariate prognostic impact. We observed that lymph node disease (N-Stage) was significantly associated with the ratio of tumor/normal (T/N) triplex-binding activity for cytoplasmic and nuclear extracts and total values (p = 0.026; 0.019; 0.017, respectively, Table 2a). This meant that all patients without lymph node disease at diagnosis had significantly decreased binding ratios (T/N) in both cytoplasmic and nuclear extracts. Also, the triplex DNA-binding activity in tumor nuclear extracts and total tumor extracts correlated significantly with metastasis (p = 0.031, p = 0.046, respectively, Table 2b). Kaplan-Meier survival analysis using a median cut-off of 1.5 (rounded-up) for the nuclear binding activity ratio (T/N) showed significantly lower overall survival in patients whose T/N nuclear binding activity ratio was greater than 1.5 (n = 30; p = 0.026) than in patients whose ratio was less than 1.5 (n = 33, Figure 3, Additional file 2). This suggested that although triplex DNA-binding protein(s) were present in normal colorectal tissue extracts, they were more abundant in tumor extracts. It also suggested that an abundance of the major triplex-binding EMSA complex (H3) in the nuclei of tumor cells was associated with metastasis and reduced overall survival (Additional file 3). 

**Table 2:** Correlation of the ratio of tumor (T) to normal (N) (T/N) EMSA H3 values for each patient with clinical features: test statistics (a) by presence of disease in lymph nodes (N-Stage) and (b) by presence of metastasis in distant organs (distant metastasis)


**(a) Grouping Variable: presence of disease in lymph nodes (N Stage) dichotomized**			
	**Ratio T/N**	**Ratio T/N**	**Ratio T/N**
	**cytoplasm**	**nucleus**	**total**
**Mann-Whitney U**	**322.000**	**313.000**	**310.500**
**Wilcoxon W**	**1025.000**	**1016.000**	**1013.500**
**Z**	**-2.220**	**-2.345**	**-2.380**
**Asymp. Sig (2-tailed)**	**0.026**	**0.019**	**0.017**
**(b) Grouping Variable: presence of metastasis in distant organs (distant metastasis)**			
	**Cytoplasm tumor**	**Nucleus tumor**	**Total tumor**
**Mann-Whitney U**	**342.500**	**309.500**	**321.000**
**Wilcoxon W**	**1245.500**	**1212.500**	**1224.000**
**Z**	**-1.689**	**-2.156**	**-1.993**
**Asymp. Sig (2-tailed)**	**0.091**	**0.031**	**0.046**

### Identification of U2AF65 as the protein present in the EMSA H3 complex

We wished to identify the protein(s) responsible for binding the triplex DNA probe in the major EMSA H3 complex. We isolated biotinylated purine-motif triplex DNA-protein complexes from RKO cells with streptavidin-conjugated agarose, separated the complexes by SDS-PAGE, and stained with Coomassie Blue. Protein bands were analyzed by nano-HPLC ESI-MS-MS and identified using MASCOT database searches. We identified (1) 100-kDa and (2) 60-kDa proteins from nuclear extracts and a (3) 65-kDa protein from cytoplasmic extracts. These corresponded to the following proteins:

(1) PSF (polypyrimidine tract binding-associated splicing factor, or SFPQ) [NCBI Protein AAH04534]

(2) P54nrb (nuclear RNA-binding protein) or NonO [NCBI Protein NP_031389]

(3) U2AF65 (U2 small nuclear RNA auxiliary factor 2 isoform b) [NCBI Protein NP_001012496]

PSF and p54nrb are known to function as RNA polymerase II-associated splicing factors, bind as heterodimers, and are implicated in the regulation of expression of the Myc family of oncoproteins, COX2, etc. They also bind to and stimulate topoisomerase I and promote homologous DNA pairing and the incorporation of a single-stranded oligonucleotide into homologous superhelical double-stranded DNA D-loop formation [33,34]. U2AF65, identified from cytoplasmic extracts, is also an RNA polymerase II-associated splicing factor that can associate with mRNAs that include a predominance of transcription factors and cell cycle regulators, and shuttle continuously between the nucleus and cytoplasm [35,36].

**Figure 4:**
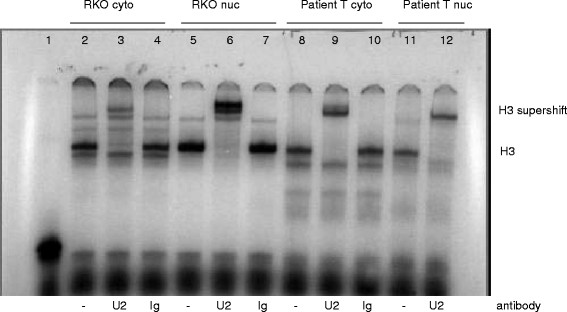
**Production of a super-shifted H3 band in RKO and patient tissue extracts by super-shift EMSA with a monoclonal antibody against U2AF65. **^33^P-labeled triplex DNA (1 nM) was complexed with 1.5 μg total protein from RKO cytoplasmic (lanes 2-4), RKO nuclear (lanes 5-7), 5 μg tumor cytoplasm (T cyto lanes 8-10) or tumor nuclear (T nuc lanes 11-12) extracts. Lanes 2, 5, 8, and 11, no antibody; lanes 3, 6, 9, and 12, 400 ng anti-U2AF65 antibody MC3; lanes 4, 7, and 10, mouse IgG antibody (negative control). Each reaction also contained 2 μg poly (dI-dC) carrier DNA. Lane 1, triplex DNA probe alone.

Super-shift EMSA with a well-characterized monoclonal antibody against U2AF65 [37] consistently produced a super-shifted H3 band in all human extracts tested that were known to express U2AF65 by Western blot analysis (RKO and tumor tissue cytoplasmic and nuclear extracts are shown in Figure 4). This confirmed that U2AF65 is present in the H3 triplex DNA-protein complex observed by EMSA (Figure 4). Available antibodies against PSF or p54nrb did not produce any super-shifted bands in our EMSA analysis (Additional file 1: Figure S3). 

### U2AF65 expression correlates with EMSA H3 values and p54nrb and PSF expression in tumor tissues and with a higher tumor stage

We measured expression of the three splicing factors in normal and tumor colorectal tissue extracts obtained from 51 of the 63 patients using Western blotting to determine if triplex DNA-binding activity in EMSA correlates directly with U2AF65, PSF, and/or p54nrb total protein expression. Spearman correlations indicated that U2AF65 expression correlated significantly with EMSA H3 values, and that the correlation was highly significant in tumor extracts (cytoplasmic p = 1.8e-8; nuclear p = 5.9e-5; total p = 1.8e-8; Table 3a, Additional file 4). In comparison, PSF and p54nrb were highly expressed in nuclear extracts but seldom detected in cytoplasmic extracts, and their expression correlated with EMSA H3 values only in tumor nuclear extracts (p = 0.036 and 0.0071, respectively) (Table 3a). When correlating the expressions of the three splicing factors with each other, PSF and p54nrb were highly significantly associated in nuclear extracts of both normal and tumor tissue (p = 1e-6 in both) as expected, as they are known to bind and function as heterodimers. Also, U2AF65 expression was highly significantly correlated with p54nrb expression in both normal and tumor nuclear extracts (p = 0.00037 and 1e-6, respectively)(Table 3b), but with PSF expression only in tumor nuclear extracts (p = 0.0005), suggesting a unique functional aspect of U2AF65 and PSF in tumor cell nuclei. We also examined expression of the three splicing factors identified by biotin triplex DNA affinity in the eight colorectal cancer cell lines using Western blotting. Consistent with patient tissue data, U2AF65 expression from all cell line extracts most closely matched the abundance of the EMSA H3 band, with moderate expression in all cytoplasmic extracts and abundant expression in all nuclear extracts (Additional file 1: Figure S2b).

**Table 3:** (a) Spearman correlation p values of EMSA H3 values with expression of triplex DNA-binding proteins (3BP) and (b) correlations of U2AF65 expression to PSF and p54nrb expression in normal and tumor tissue extracts

**3BP expression correlated**	**U2AF65**	**p54nrb**	**PSF**
**With EMSA H3**			
**Normal cytoplasm**	**0.0028**	**0.91**	**0.66**
**Normal nucleus**	**0.015**	**0.11**	**0.075**
**Normal total**	**0.016**	**0.26**	**0.095**
**Tumor cytoplasm**	**1.8e-08**	**0.53**	**0.019**
**Tumor nucleus**	**5.9e-05**	**0.0071**	**0.036**
**Tumor total**	**1.8e-08**	**0.0048**	**7.5e-05**
**Correlation to U2AF65**	**p54nrb**	**PSF**	
**Normal cytoplasm**	**0.00066**	**0.085**	
**Normal nucleus**	**0.00037**	**0.073**	
**Normal total**	**0.00040**	**0.094**	
**Tumor cytoplasm**	**0.0041**	**0.0002**	
**Tumor nucleus**	**1e-06**	**0.0005**	
**Tumor total**	**1e-06**	**1e-06**	

**Figure 5:**
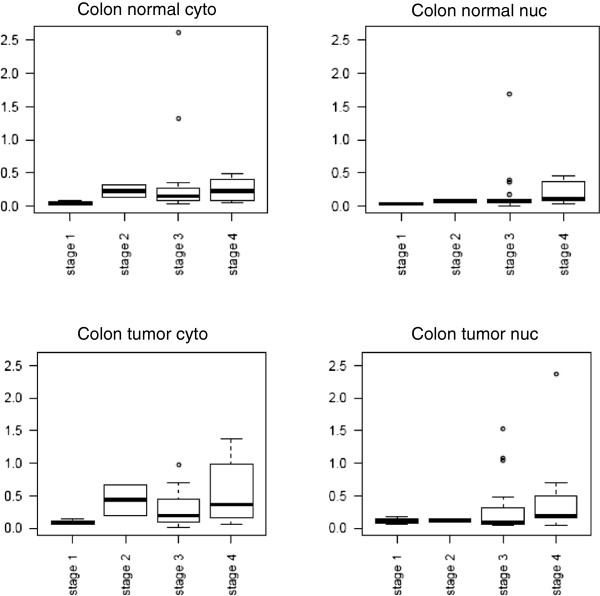
**U2AF65 protein expression by colon tumor stage. **Total protein (25 μg) from cytoplasmic (cyto) and nuclear (nuc) colon tumor and normal tissue extracts were separated using 10% SDS-PAGE and electro-transferred to nitrocellulose membranes. Blots were incubated with anti-U2AF65 antibody MC-3 and detected using chemiluminescence and autoradiography. Blots were reprobed with an anti-actin antibody, and densitometry was performed using NIH Image J software. U2AF65 expression values were normalized by dividing the actin expression values in each extract, and plotted according to colon tumor stage using the R program (Additional file 6).

Having shown that the EMSA H3 complex was increased in tumor compared to adjacent normal tissue, we wished to determine if U2AF65, p54nrb and PSF expression was associated with tumor stage. U2AF65 protein expression according to extract type and tumor stage in all colon tumors is shown in Figure 5. Colon tumors in Figure 5 in advanced clinical stages, UICC Stage III and IV (Dukes C and D) express significantly higher U2AF65 in the cytoplasm and overall than did tumors at early stages (mean value of U2AF65 tumor cytoplasm UICC Stage I and II expression = 0.349 vs. UICC Stage III and IV = 0.491; p = 0.024 [Mann-Whitney *U*-Test, Additional file 5]). PSF and p54nrb expression were not significantly correlated with tumor stage. While both p54nrb and PSF expression were significantly correlated with EMSA H3 values in tumor but not normal tissue extracts, the antibodies against these proteins that we tested were unable to produce a super-shifted EMSA band. Thus the relevance of p54nrb and PSF as triplex DNA-binding proteins remains to be determined.

### Expression of the WRN helicase correlates with EMSA H3 binding activity

We wanted to test the hypothesis that proteins that bind to or stabilize triplexes and G-quadruplexes can act in a yin-yang fashion (in complementary opposition) with proteins such as helicases that unwind or destabilize these structures, and that expression and/or function of these binding and unwinding proteins may be imbalanced in tumors that could contribute to genomic instability. We tested 51 patient colorectal tumor and normal tissue extracts for expression of the RecQ-family helicase WRN because it is known to act preferentially on aberrant structures such as triplexes and G-quadruplexes and to promote genomic integrity [19]. We used the Wilcoxon sign rank test to determine if WRN is differentially expressed in normal and tumor tissue extracts and Spearman’s rho to correlate WRN helicase expression in normal and tumor tissue extracts with EMSA H3 data. We detected no significant differences in normalized WRN expression between normal and tumor extracts or according to tumor stage (mean cytoplasmic expression in tumor tissue = 0.424, in normal tissue = 0.283; mean nuclear expression in tumor tissue = 0.275, in normal tissue = 0.196; total expression mean in tumor tissue = 0.679, in normal tissue = 0.465). However, we did observe that total WRN expression correlated significantly with total EMSA H3 binding values in both normal tissue (rho 0.296, p = 0.03) and tumor extracts (rho 0.460, p < 0.001).

### Reverse-phase protein array and western blot analysis of tissue extracts show a correlation of U2AF65 expression with total and truncated beta-catenin expression

Another goal of our study was to measure the expression of numerous cancer-relevant proteins in patient tissue extracts and correlate it with EMSA H3 values and expression of the three splicing factors identified using biotin triplex DNA affinity as a screen to identify potentially relevant functional relationships among these splicing factors and other well-characterized proteins. Using reverse-phase protein array (RPPA) analysis, we examined extracts from 51 patients (because not all extracts met the minimum concentration needed for accurate measurement) for expression of cancer-related proteins with 37 previously validated antibodies. Spearman correlation of the expression of multiple signaling proteins was calculated. Significant correlations after Bonferroni correction for multiple testing were found with both EMSA H3 values and U2AF65 expression, including NF- B p65, GSK3 beta, beta-catenin, Src, and PI3K p110 alpha (Table 4; exact p values are shown in Additional file 7: Table S1). The expression levels of a distinct set of proteins were found to correlate significantly with both p54nrb and PSF expression, such as cyclin D1, c-Myc, JNK1, CDK4, Akt1, and Stat3. Expression of all three splicing factors and EMSA H3 values also significantly correlated with another set of proteins including p38 alpha, ErbB1 (EGFR), mTOR, PTEN, and Stat5.

**Table 4:** Spearman correlations of EMSA H3 values and triplex DNA-binding protein expression to other proteins by reverse phase protein array (RPPA)

	**EMSAH3**	**U2AF65**	**p54nrb**	**PSF**
**Ph-Erk**		**		
**NF-κB p65**	***	***		
**Cyclin D1**			***	***
**GSK3β**	***	*		
**c-myc**			**	***
**JNK1**			***	***
**PCNA**	***	***		**
**β-catenin**	***	***		
**Ph-Raf**				**
**Src**	**	**		
**p 38 α**	**	**	***	***
**Cdk4**			**	***
**Akt1**			**	***
**ErbB1**	***	***	**	***
**Bcl-2**		**		
**mTor**	***	***	***	***
**P13K p110 α**	**	***		
**PLC γ**				***
**PTEN**	***	***	***	***
**Stat3**	*		**	***
**Stat5**	***	***	***	***

*p < 0.05; **p < 0.01; ***p < 0.001.

**Figure 6:**
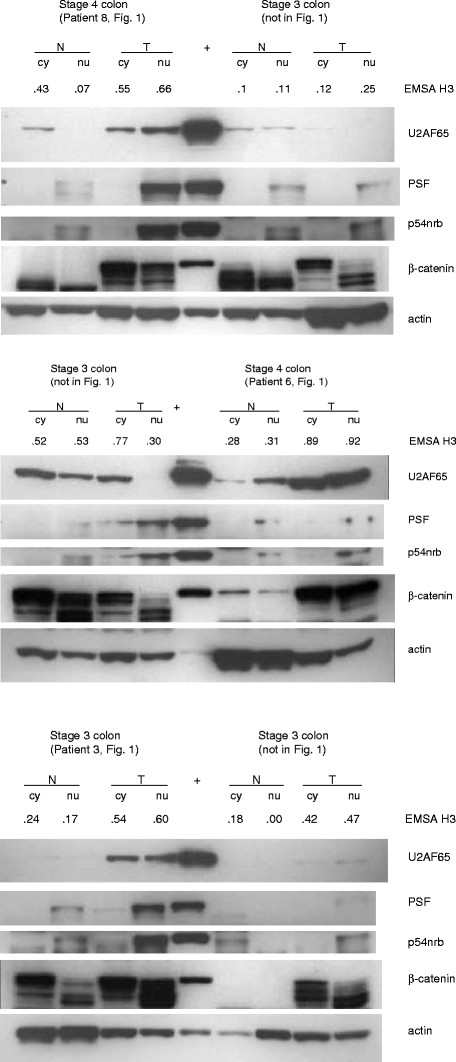
**Western blots of U2AF65, PSF, p54nrb, and beta-catenin expression in normal and tumor colorectal tissue extracts. **Total protein (25 μg) from cytoplasmic (cy) and nuclear (nu) tissue extracts obtained from six selected patients were separated using 10% SDS-PAGE and electro-transferred to nitrocellulose membranes. Blots were incubated with the antibodies against U2AF65, PSF, p54nrb, beta-catenin, and actin, then the appropriate secondary antibody and detected using chemiluminescence and autoradiography. Each patient’s tumor stage and number which are also included in Figure 1, and corresponding EMSA H3 values are shown above the samples.

The most highly significant correlation in our RPPA analysis was that between U2AF65 expression and beta-catenin (p = 9e-10), known to be deregulated and a major player in the etiology of colorectal cancer. To confirm our RPPA results, we compared Western blots of beta-catenin and U2AF65 expression in tissue extracts from 50 patients. Representative Western blots for six patients are shown in Figure 6, which includes some patient samples also shown in Figure 1 EMSAs. These data were quantitated by densitometry and graphed in Additional file 1: Figure S4. According to Spearman’s rho, we observed that total beta-catenin and U2AF65 expression are highly significantly correlated in cytoplasmic and nuclear tumor extracts (p = 5.7e-6 and p = 3.1e-6, respectively), while their expression correlated significantly in normal nuclear extracts (p = 0.0018), and showed no significant correlation in normal cytoplasmic extracts (p = 0.15). In addition, beta-catenin expression was higher in cytoplasmic and nuclear extracts of stage III and IV colon tumors than in those of stage I and II colon tumors (Additional file 1: Figure S5). Western blots of beta-catenin expression showed truncated bands (65-80- kDa) for some extracts but not for others, which was consistent with previous reports of truncated or novel spliceforms of beta-catenin mRNA [38,39] and an 80-kDa truncated beta-catenin protein [40] in colorectal cancer. In addition to a significant correlation between full-length beta-catenin (92-kDa) expression and U2AF65 expression, we found a significant correlation between truncated beta-catenin and U2AF65 expression, particularly in the cytoplasm (p = 0.0047) and nuclei (p = 0.022) of tumor cells. 

## Discussion

The data provides support to the hypothesis that the major triplex DNA-binding protein in human cells is more abundant and has higher binding activity *in vitro* in extracts from colorectal cancer tissues compared to adjacent normal tissues. This increased binding activity correlated significantly with the expression of triplex/G-quadruplex DNA-unwinding helicase WRN, and with the spread of cancer to the lymph nodes, metastasis, and reduced overall survival. The major triplex DNA-binding protein in gel shifts was identified as the U2AF65 splicing factor. U2AF65 expression was higher in more advanced colon tumor stages and correlated significantly with total and truncated beta-catenin expression.

U2AF is a non-small nuclear ribonucleoprotein (snRNP) splicing factor required for the binding of U2 snRNP to the pre-mRNA branch site [41,42]. Purified U2AF is comprised of two polypeptides of 65- (U2AF65) and 35-kDa (U2AF35), respectively. U2AF65 binds to the polypyrimidine (Py) tract adjacent to the 3’ splice site using RNA-recognition motifs and cross-links to the branch point in an ATP-independent manner at the earliest stage of spliceosome formation [43]. Both subunits of U2AF are essential for the viability of many model organisms, such as zebra fish, *Drosophila*, *C. elegans*, and *S. pombe*[44]. Both U2AF65 and U2AF35 shuttle continuously between the nucleus and cytoplasm by a mechanism that involves carrier receptors and is independent from binding to mRNA. It has also been suggested that U2AF participates in the nuclear export of mRNA [45].

U2AF65 binds to single-stranded RNA and recognizes a wide variety of pyrimidine (Py)-tracts. The Py-tracts of higher eukaryotic pre-mRNAs are often interrupted with purines, yet U2AF65 must identify these degenerate Py-tracts for accurate pre-mRNA splicing. Based on *in vitro* studies, investigators have proposed that U2AF35 assists U2AF65 recruitment to nonconsensus polypyrimidine tracts. Pacheco *et al.* analyzed the roles of the two U2AF subunits *in vivo* in the selection of alternative 3' splice sites associated with polypyrimidine tracts of different strengths. Their results revealed a feedback mechanism by which RNA interference-mediated depletion of U2AF65 triggers down regulation of U2AF35 expression. They also showed that knockdown of each U2AF subunit inhibits weak 3' splice site recognition, while over-expression of U2AF65 alone is sufficient to activate selection of this splice site [46,47]. It would be interesting to examine if over-expression of U2AF65 alone in the context of cancer activates splicing of weak or nonconsensus polypyrimidine tracts that could tip the balance of splicing regulation in a subset of cellular transcripts which could promote tumorigenesis.

The proteins we identified in RKO nuclear extracts using biotin triplex DNA affinity were PSF, a 100-kDa protein that also binds to the polypyrimidine tract, and its heterodimeric binding partner p54nrb. We speculate that the 100- and 60-kDa proteins identified in previous studies using Southwestern blotting with HeLa nuclear extracts [16] probed with the same purine triplex DNA probe used in this study are indeed PSF and p54nrb, but this has yet to be tested. Both PSF and p54nrb bind to double-stranded (ds)DNA, single-stranded (ss)DNA, and RNA, and contain DNA- and RNA-binding domains. PSF participates in constitutive pre-mRNA splicing and is a component of later spliceosomal B and C complexes (when U2AF65 is no longer present). PSF and p54nrb also bind and function in nuclear retention of defective RNAs and are involved in transcriptional regulation and the DNA damage response [48-51]. Interestingly, PSF also functions in DNA annealing, where PSF requires ssDNA and dsDNA with sequence homology for their *in vitro* pairing activity as well as divalent cations. PSF can promote the incorporation of ssDNA within the two separated strands of a homologous superhelical DNA duplex and produce a three-stranded D-loop structure, which is required for homologous recombination. Other splicing factors SF2/ASF and U2AF65 also caused DNA annealing but could not form D loops [52]. PSF and p54nrb, as well as GRSF-1, YB-1, and polypyrimidine tract-binding protein (PTB) also bind to the *MYC* family of internal ribosome entry sites (IRES) and positively regulate translation of the Myc family of oncoproteins *in vitro* and *in vivo*[53]. Protein array data in this study showed that expression of both PSF and p54nrb in colorectal tissue extracts correlated significantly with c-Myc expression levels, which is consistent with a role for PSF and p54nrb in the regulation of c-Myc protein expression.

Researchers identified both U2AF and PSF, as well as hnRNP C and PTB, as RNA-binding proteins that bind to two regions 3’ of the (CUG)_n_ repeat expansion in the 3’-UTR of the *DMPK* gene, where expansion of this trinucleotide repeat causes the neuromuscular disorder myotonic dystrophy [54]. Their study explored RNA-binding proteins interacting with non-CUG regions or higher order structures in the *DMPK* 3’-UTR that may be involved in RNA-mediated pathogenesis. Their finding that both U2AF and PSF can bind near this triplet repeat sequence with the potential to form higher order structures such as triplexes is consistent with our data on biotin triplex DNA affinity identification of both U2AF65 and PSF. Another group identified an RNA/protein complex in both *Drosophila* and 293 cells that consisted of expanded CAG RNA, U2AF65, and the NXF1 nuclear export receptor, providing further evidence that in other models, U2AF65 interacts with these triplet repeat sequences [55]. We believe that the purine triplex DNA EMSA probe can be a surrogate multiplex nucleic acid structure that acts as a “bait and hook” to capture proteins that may be binding D-loops, R-loops, triplexes, G-quadruplexes, or other multi-stranded structures containing Hoogsteen or reverse Hoogsteen base pairs *in vivo*.

PTB also binds to polypyrimidine tracts in pre-mRNAs, and numerous studies have shown that PTB competes with U2AF65 for binding to these sequences [56-61]. Since PSF is a PTB-associated protein, binding competition between PSF and U2AF65 may be possible as well, which may explain why we identified both PSF with the biotinylated triplex DNA in RKO nuclear extracts and U2AF65 in RKO cytoplasmic extracts. Gama-Carvalho and colleagues performed immunoprecipitation of U2AF65- and PTB-associated RNAs from HeLa cells followed by microarray analysis to determine which mRNAs are associated with these two splicing factors that can compete for binding to polypyrimidine tracts [36]. Among U2AF65-associated mRNAs was a predominance of transcription factors and cell cycle regulators, whereas PTB-associated transcripts were enriched in mRNAs that encode proteins implicated in intracellular transport, vesicle trafficking, and apoptosis.

Related to cancer, researchers found that 2 of 14 patients with malignant mesothelioma, a pulmonary malignancy, had antibodies against U2AF65 using the SEREX technique (serologic identification by recombinant expression cloning) [62]. Additionally, a patient with liver cirrhosis that progressed to hepatocellular carcinoma had antinuclear antibodies that recognized a nuclear protein putatively identified as U2AF65 [63]. Other splicing factors, most notably SFRS1 (ASF/SF2), are reported to be over-expressed in colon, thyroid, kidney, lung and breast cancer cells [64]. Other splicing factors shown to be over-expressed in colorectal cancer cells are hnRNP-F and –K, SPF45, and SRPK1 [64]. However, the present report is the first to describe correlation of increased expression or binding activity of U2AF65 in primary colorectal tumors with tumor stage, lymph node disease, metastasis and reduced overall survival.

Why U2AF65 is over-expressed in colorectal tumor cells, and whether this over-expression is important to the development and/or progression of colorectal cancer or a passive effect of general gene deregulation are unknown. About 75% of sporadic colorectal cancers are characterized by a chromosomal instability (CIN) phenotype. The most common reported chromosomal losses involve 5q (APC), 18q (DCC), and 17p (p53), while the most common gains involve 8q and 20q. The gene encoding U2AF65 (*U2AF2*) is located at c19q13.42. Chromosomal amplifications at c19q13.42 have been found in a rare embryonal tumor using array CGH and FISH [65,66]. Other groups have reported amplifications or aberrations at c19q13 in colorectal tumors, particularly in liver metastases compared to primary tumors [67], and in other solid tumors including pancreatic [68] and ovarian [69].

Regarding genomic instability, Vasquez and colleagues recently showed that both non-B DNA sequences and WRN helicase deficiency induce mutations characterized by single base changes, mostly at C-G base pairs, in an additive but not synergistic manner [70]. Because no synergy was observed, the authors concluded that a role for WRN in reducing mutation frequencies via a mechanism dependent on its cellular helicase activity (for example, of non-B DNA sequences) is unlikely. Their data do not directly support our present hypothesis, which is similar to their hypothesis that if one function of the WRN helicase were to resolve non-B (triplex and Z-DNA) structures, as observed *in vitro*, then mutation frequencies may be higher in WRN-deficient cells than in WRN-wild type cells because both the number and stability of such structures would be greater in WRN-deficient cells. However, they did verify that purified WRN protein was able to unwind the third purine-rich strand of a synthetic triplex *in vitro*. Although our data suggest a correlation between expression of the WRN helicase with triplex DNA-binding activity in both normal and tumor tissue extracts, defining a functional role and mechanism of non-B DNA unwinding activity by WRN helicase and G*G multiplex binding (for example, by U2AF65) will require further study.

Beta-catenin, as a transcription factor complexed with TCF4, is known to upregulate expression of many relevant proteins in colorectal cancer, such as c-myc, cyclin D1, LEF-1, CD44, and c-jun. Whether beta-catenin influences the expression of U2AF65 is unknown, but a search of transcription factor binding sites in the U2AF65 (*U2AF2*) gene promoter did not indicate any beta-catenin or TCF family transcription factor sites among the 55 high-scoring (>85%) sites we identified (Cold Spring Harbor Laboratory Mammalian Promoter Database http://rulai.cshl.edu/CSHLmpd2/; Transcription Factor Search http://www.cbrc.jp/research/db/TFSEARCH.html). Similarly, mining through microarray expression studies revealed no reports describing U2AF65 (*U2AF2*) as a beta-catenin, TCF4, or Wnt target gene (NCBI GEO; R Nusse Wnt/Beta catenin targets list: http://www.stanford.edu/~rnusse/pathways/targets.html). The biological significance of the correlation of U2AF65 and beta-catenin expression in colorectal tumor tissues, such as if beta-catenin as a transcription factor affects U2AF65 expression, or if U2AF65 as a splicing factor affects the splicing or expression of beta-catenin, remains to be determined.

Several studies have examined the interaction of beta-catenin with splicing factors and the role of beta-catenin in mRNA splicing. Researchers identified alternative splicing of SLC39A14, a divalent cation transporter, in colorectal tumors and found it to be regulated by the Wnt pathway, probably through regulation of splicing factor SRSF1 [71]. The beta-catenin/TCF4 pathway also modifies alternative splicing through modulation of expression of splicing factors SRp20 [72] and SF1 [73] and direct interaction with FUS/TLS (translocated in liposarcoma) and various other RNA-binding proteins, including p54nrb [74]. Others have shown that beta-catenin regulates multiple steps of RNA metabolism in colon cancer cells and may coordinate RNA metabolism [75].

Authors have also reported identification of truncated beta-catenin isoforms, mostly in colorectal cancer cells. In primary colorectal tumors, a relatively small percent (7 of 58 examined) contained somatic interstitial deletions that included all or part of exon 3 of the beta-catenin gene, and RT-PCR analysis from 3 of the 7 tumors detected transcripts that lacked exon 3 and the presence of the normal transcript [39]. Researchers also detected two novel beta-catenin mRNA splice variants in the SW480 colon cancer cell line and in primary colorectal tumors [38]. A truncated beta-catenin protein of 80-kDa was also detected in three colorectal metastases to the liver [40]. Several of these isoforms have truncations in the NH_2_-terminus of the protein that produce deletions of key serine and threonines that are phosphorylated by GSK-3 beta, which is important for proteosomal degradation, which was hypothesized to stabilize the protein and have a dominant oncogenic effect [76]. Data from this and other studies lead us to speculate that U2AF65 could be binding to a multi-stranded nucleic acid structure such as R-loops, D-loops, or G-quartet mRNA *in vivo* that is mimicked by the purine triplex DNA probe in our study, and that overexpression or increased EMSA binding activity of U2AF65 in tumor tissues could cause deregulation of mRNA splicing and protein isoform expression, such as beta-catenin, that could contribute to colorectal cancer initiation and/or progression.

## Conclusions

We found that increased triplex DNA-binding activity in colorectal tumor extracts *in vitro* is associated with WRN helicase expression, increased total beta-catenin expression, lymph node disease, metastasis, and reduced overall survival in patients with colorectal cancer. Multifunctional splicing factor U2AF65 was identified as the major triplex-binding protein in human tissues and cell lines. Increased expression of U2AF65 is also associated with expression of splicing factors PSF and p54nrb, a higher tumor stage, and increased truncation of beta-catenin in colorectal tumors. We believe that our results contribute to and generate interest in the growing fields of alternative non-B DNA structures and genomic instability, aberrantly regulated splicing factors, mRNA splicing and protein isoforms related to cancer both as basic research objectives regarding the etiology of cancer and cancer diversity and as novel translational research in the search for promising prognostic, diagnostic and targeting tools.

## Competing interests

The authors declare that they have no competing interests.

## Authors’ contributions

LDN, HM, MWVD and HA designed the study; CB, DB, PK, and GM performed all statistical analysis and collected patient clinical data; LDN and HM performed all experiments; UK supervised reverse phase protein array experiments; LDN, DPH, MWVD and HA wrote the manuscript. All authors read and approved the final manuscript.

## Supplementary Material

Additional file 1 **Figure S1. **Electrophoretic Mobility Shift Assay (EMSA) of patient tissue lysates and HeLa nuclear extract with triplex and parent duplex DNA probes. ^33^P‐labeled purine‐motif duplex or triplex DNA (1 nM) was complexed with 5 μg protein from normal tissue cytoplasmic (N cy), normal nuclear (N nu), tumor tissue cytoplasmic (T cy) or tumor nuclear (T nu) extracts of colorectal cancer patients. 1.25 μg HeLa nuclear extract (H) was used as a control in lanes 6 and 12. Purine triplex probe alone is in lane 1 and duplex probe alone is in lane 7. **Figure S2a.** Electrophoretic Mobility Shift Assay (EMSA) of Cytoplasmic and Nuclear Extracts from Eight Colorectal Cancer Cell Lines with Purine triplex DNA. ^33^P‐labeled purine‐motif triplex DNA (1 nM) was complexed with 1.25 μg total protein from cytoplasmic (cy) or nuclear (nuc) extracts from eight colorectal cancer cell lines. 1.25 μg HeLa cytoplasmic and nuclear extracts were used as positive (+) controls. Each reaction also contained 2 μg poly (dI‐dC) carrier DNA. The purine triplex DNA probe alone is shown in lane 1. **Figure S2b.** Western blots showing expression of three candidate triplex DNA‐binding proteins in eight colorectal cancer cell lines. Total protein (25 μg) from cytoplasmic (cy) and nuclear (nu) extracts from eight colorectal cancer cell lines were separated using 10% SDS‐PAGE and electro‐transferred to nitrocellulose membranes. Blots were incubated with the antibodies against PSF, U2AF65, p54nrb, beta‐catenin, and actin, then the appropriate secondary antibody and detected using chemiluminescence and autoradiography. **Figure S3.** Lack of a super‐shifted H3 band in RKO nuclear extract by super‐shift EMSA with antibodies against PSF and p54nrb. ^33^P‐labeled triplex DNA (1 nM) was complexed with 1.5 μg total protein from RKO nuclear extracts (lanes 2‐9). Lane 1, triplex DNA probe alone; Lane 2, no antibody; lane 3, 400 ng anti‐U2AF65 antibody MC3; lane 4, 1000 ng anti‐U2AF65 antibody MC3; lane 5, 400 ng anti‐PSF antibody; lane 6 1000 ng anti‐PSF antibody; lane 7, 400 ng anti‐p54nrb antibody; lane 8, 1000 ng anti‐p54nrb antibody; lane 9, mouse IgG antibody (negative control). Each reaction also contained 2 μg poly (dI‐dC) carrier DNA. **Figure S4.** Quantitation of Protein Expression of PSF, U2AF65, p54nrb, and beta‐catenin obtained from six colorectal cancer patients’ tissue extracts. Autoradiographs from Western blots in Figure 6 were scanned, and protein expression bands were quantitated using NIH Image J. Protein expression was normalized by dividing by the samples’ corresponding actin value and graphed using Graph Pad. **Figure S5.** Beta‐catenin Expression by Tumor type and Stage. Western blots using an anti‐beta‐catenin antibody to examine expression in patient extracts were described for Figure 6. Beta‐catenin expression values were normalized by dividing the actin expression value in each extract, and plotted according to colon or rectum tumor stage using the R program. N cyto, cytoplasmic normal tissue extracts; N nuc, nuclear normal tissue extracts; T cyto, cytoplasmic tumor tissue extracts; T nuc, nuclear tumor tissue extracts. Click here for file

Additional file 2 DB-Triplexdata. Click here for file

Additional file 3 PK Statistical analysis Triplex. Click here for file

Additional file 4 DBuergy Correlations(1). Click here for file

Additional file 5 Daniel Apr 5(1). Click here for file

Additional file 6 histograms_proteins_groups. Click here for file

Additional file 7 **Table S1. **RPPA antibodies and Spearman correlation p values. Click here for file

## References

[B1] Frank-Kamenetskii MD Mirkin SM Triplex DNA structures Annu Rev Biochem 1995 64 65 95 10.1146/annurev.bi.64.070195.000433 7574496

[B2] Patel DJ Phan AT Kuryavyi V Human telomere, oncogenic promoter and 5'-UTR G-quadruplexes: diverse higher order DNA and RNA targets for cancer therapeutics Nucleic Acids Res 2007 35 7429 7455 10.1093/nar/gkm711 17913750 PMC2190718

[B3] Belotserkovskii BP De Silva E Tornaletti S Wang G Vasquez KM Hanawalt PC A triplex-forming sequence from the human c-MYC promoter interferes with DNA transcription J Biol Chem 2007 282 32433 32441 10.1074/jbc.M704618200 17785457

[B4] Nikolova EN Kim E Wise AA O'Brien PJ Andricioaei I Al-Hashimi HM Transient Hoogsteen base pairs in canonical duplex DNA Nature 2011 470 498 502 10.1038/nature09775 21270796 PMC3074620

[B5] Hanahan D Weinberg RA Hallmarks of cancer: the next generation Cell 2011 144 646 674 10.1016/j.cell.2011.02.013 21376230

[B6] Bacolla A Wells RD Non-B DNA conformations as determinants of mutagenesis and human disease Mol Carcinog 2009 48 273 285 10.1002/mc.20507 19306308

[B7] Mirkin SM Expandable DNA repeats and human disease Nature 2007 447 932 940 10.1038/nature05977 17581576

[B8] Napierala M Bacolla A Wells RD Increased negative superhelical density in vivo enhances the genetic instability of triplet repeat sequences J Biol Chem 2005 280 37366 37376 10.1074/jbc.M508065200 16166072

[B9] Zhao J Bacolla A Wang G Vasquez KM Non-B DNA structure-induced genetic instability and evolution Cell Mol Life Sci 2010 67 43 62 10.1007/s00018-009-0131-2 19727556 PMC3017512

[B10] Jain A Wang G Vasquez KM DNA triple helices: biological consequences and therapeutic potential Biochimie 2008 90 1117 1130 10.1016/j.biochi.2008.02.011 18331847 PMC2586808

[B11] Bacolla A Collins JR Gold B Chuzhanova N Yi M Stephens RM Stefanov S Olsh A Jakupciak JP Dean M Long homopurine*homopyrimidine sequences are characteristic of genes expressed in brain and the pseudoautosomal region Nucleic Acids Res 2006 34 2663 2675 10.1093/nar/gkl354 16714445 PMC1464109

[B12] Joos S Haluska FG Falk MH Henglein B Hameister H Croce CM Bornkamm GW Mapping chromosomal breakpoints of Burkitt's t(8;14) translocations far upstream of c-myc Cancer Res 1992 52 6547 6552 1330296

[B13] Wang G Vasquez KM Naturally occurring H-DNA-forming sequences are mutagenic in mammalian cells Proc Natl Acad Sci U S A 2004 101 13448 13453 10.1073/pnas.0405116101 15342911 PMC518777

[B14] Cer RZ Bruce KH Mudunuri US Yi M Volfovsky N Luke BT Bacolla A Collins JR Stephens RM Non-B DB: a database of predicted non-B DNA-forming motifs in mammalian genomes Nucleic Acids Res 2011 39 D383 D391 10.1093/nar/gkq1170 21097885 PMC3013731

[B15] Darnell JC Jensen KB Jin P Brown V Warren ST Darnell RB Fragile X mental retardation protein targets G quartet mRNAs important for neuronal function Cell 2001 107 489 499 10.1016/S0092-8674(01)00566-9 11719189

[B16] Musso M Nelson LD Van Dyke MW Characterization of purine-motif triplex DNA-binding proteins in HeLa extracts Biochemistry 1998 37 3086 3095 10.1021/bi9717486 9485462

[B17] Nelson LD Musso M Van Dyke MW The Yeast STM1 Gene Encodes a Purine Motif Triple Helical DNA-binding Protein J Biol Chem 2000 275 5573 5581 10.1074/jbc.275.8.5573 10681538

[B18] Van Dyke MW Nelson LD Weilbaecher RG Mehta DV Stm1p, a G4 Quadruplex and Purine Motif Triplex Nucleic Acid-binding Protein, Interacts with Ribosomes and Subtelomeric Y' DNA in Saccharomyces cerevisiae J Biol Chem 2004 279 24323 24333 10.1074/jbc.M401981200 15044472

[B19] Frantz JD Gilbert W A yeast gene product, G4p2, with a specific affinity for quadruplex nucleic acids J Biol Chem 1995 270 9413 9419 10.1074/jbc.270.16.9413 7721866

[B20] Ozgenc A Loeb LA Current advances in unraveling the function of the Werner syndrome protein Mutat Res 2005 577 237 251 10.1016/j.mrfmmm.2005.03.020 15946710

[B21] Frank B Hoffmeister M Klopp N Illig T Chang-Claude J Brenner H Colorectal cancer and polymorphisms in DNA repair genes WRN, RMI1 and BLM Carcinogenesis 2010 31 442 445 10.1093/carcin/bgp293 19945966

[B22] Kawasaki T Ohnishi M Suemoto Y Kirkner GJ Liu Z Yamamoto H Loda M Fuchs CS Ogino S WRN promoter methylation possibly connects mucinous differentiation, microsatellite instability and CpG island methylator phenotype in colorectal cancer Mod Pathol 2008 21 150 158 18084250 10.1038/modpathol.3800996

[B23] Walther A Johnstone E Swanton C Midgley R Tomlinson I Kerr D Genetic prognostic and predictive markers in colorectal cancer Nat Rev Cancer 2009 9 489 499 10.1038/nrc2645 19536109

[B24] Martin ES Tonon G Sinha R Xiao Y Feng B Kimmelman AC Protopopov A Ivanova E Brennan C Montgomery K Common and distinct genomic events in sporadic colorectal cancer and diverse cancer types Cancer Res 2007 67 10736 10743 10.1158/0008-5472.CAN-07-2742 18006816

[B25] Wood LD Parsons DW Jones S Lin J Sjoblom T Leary RJ Shen D Boca SM Barber T Ptak J The genomic landscapes of human breast and colorectal cancers Science 2007 318 1108 1113 10.1126/science.1145720 17932254

[B26] Sjoblom T Jones S Wood LD Parsons DW Lin J Barber TD Mandelker D Leary RJ Ptak J Silliman N The consensus coding sequences of human breast and colorectal cancers Science 2006 314 268 274 10.1126/science.1133427 16959974

[B27] Asangani IA Rasheed SA Nikolova DA Leupold JH Colburn NH Post S Allgayer H MicroRNA-21 (miR-21) post-transcriptionally downregulates tumor suppressor Pdcd4 and stimulates invasion, intravasation and metastasis in colorectal cancer Oncogene 2008 27 2128 2136 10.1038/sj.onc.1210856 17968323

[B28] Schreiber E Matthias P Muller MM Schaffner W Rapid detection of octamer binding proteins with 'mini-extracts', prepared from a small number of cells Nucleic Acids Res 1989 17 6419 10.1093/nar/17.15.6419 2771659 PMC318318

[B29] Ausubel FM Current protocols in molecular biology 1987 Pa: Greene Pub. Associates. J. Wiley, order fulfillment, Brooklyn, N.Y. Media

[B30] Mannsperger HA Uhlmann S Schmidt C Wiemann S Sahin O Korf U RNAi-based validation of antibodies for reverse phase protein arrays Proteome Sci 2010 8 69 10.1186/1477-5956-8-69 21182776 PMC3022873

[B31] Loebke C Sueltmann H Schmidt C Henjes F Wiemann S Poustka A Korf U Infrared-based protein detection arrays for quantitative proteomics Proteomics 2007 7 558 564 10.1002/pmic.200600757 17309101

[B32] Mannsperger HA Gade S Henjes F Beissbarth T Korf U RPPanalyzer: Analysis of reverse-phase protein array data Bioinformatics 2010 26 2202 2203 10.1093/bioinformatics/btq347 20634205

[B33] Straub T Grue P Uhse A Lisby M Knudsen BR Tange TO Westergaard O Boege F The RNA-splicing factor PSF/p54 controls DNA-topoisomerase I activity by a direct interaction J Biol Chem 1998 273 26261 26264 10.1074/jbc.273.41.26261 9756848

[B34] Shi Y Di Giammartino DC Taylor D Sarkeshik A Rice WJ Yates JR Frank J Manley JL Molecular architecture of the human pre-mRNA 3' processing complex Mol Cell 2009 33 365 376 10.1016/j.molcel.2008.12.028 19217410 PMC2946185

[B35] Gama-Carvalho M Carvalho MP Kehlenbach A Valcarcel J Carmo-Fonseca M Nucleocytoplasmic Shuttling of Heterodimeric Splicing Factor U2AF J Biol Chem 2001 276 13104 13112 10.1074/jbc.M008759200 11118443

[B36] Gama-Carvalho M Barbosa-Morais NL Brodsky AS Silver PA Carmo-Fonseca M Genome-wide identification of functionally distinct subsets of cellular mRNAs associated with two nucleocytoplasmic-shuttling mammalian splicing factors Genome Biol 2006 7 R113 10.1186/gb-2006-7-11-r113 17137510 PMC1794580

[B37] Gama-Carvalho M Krauss RD Chiang L Valcarcel J Green MR Carmo-Fonseca M Targeting of U2AF65 to Sites of Active Splicing in the Nucleus J Cell Biol 1997 137 975 987 10.1083/jcb.137.5.975 9166400 PMC2136214

[B38] Pospisil H Herrmann A Butherus K Pirson S Reich JG Kemmner W Verification of predicted alternatively spliced Wnt genes reveals two new splice variants (CTNNB1 and LRP5) and altered Axin-1 expression during tumour progression BMC Genomics 2006 7 148 10.1186/1471-2164-7-148 16772034 PMC1523213

[B39] Iwao K Nakamori S Kameyama M Imaoka S Kinoshita M Fukui T Ishiguro S Nakamura Y Miyoshi Y Activation of the beta-catenin gene by interstitial deletions involving exon 3 in primary colorectal carcinomas without adenomatous polyposis coli mutations Cancer Res 1998 58 1021 1026 9500465

[B40] Hugh TJ Dillon SA O'Dowd G Getty B Pignatelli M Poston GJ Kinsella AR beta-catenin expression in primary and metastatic colorectal carcinoma Int J Cancer 1999 82 504 511 10.1002/(SICI)1097-0215(19990812)82:4<504::AID-IJC6>3.0.CO;2-6 10404062

[B41] Zamore PD Green MR Biochemical characterization of U2 snRNP auxiliary factor: an essential pre-mRNA splicing factor with a novel intranuclear distribution EMBO J 1991 10 207 214 1824937 10.1002/j.1460-2075.1991.tb07937.x PMC452631

[B42] Ruskin B Zamore PD Green MR A factor, U2AF, is required for U2 snRNP binding and splicing complex assembly Cell 1988 52 207 219 10.1016/0092-8674(88)90509-0 2963698

[B43] Gaur RK Valcarcel J Green MR Sequential recognition of the pre-mRNA branch point by U2AF65 and a novel spliceosome-associated 28-kDa protein RNA 1995 1 407 417 7493318 PMC1482402

[B44] Sridharan V Heimiller J Singh R Genomic mRNA profiling reveals compensatory mechanisms for the requirement of the essential splicing factor U2AF Mol Cell Biol 2011 31 652 661 10.1128/MCB.01000-10 21149581 PMC3028654

[B45] Zolotukhin AS Tan W Bear J Smulevitch S Felber BK U2AF participates in the binding of TAP (NXF1) to mRNA J Biol Chem 2002 277 3935 3942 10.1074/jbc.M107598200 11724776

[B46] Pacheco TR Moita LF Gomes AQ Hacohen N Carmo-Fonseca M RNA interference knockdown of hU2AF35 impairs cell cycle progression and modulates alternative splicing of Cdc25 transcripts Mol Biol Cell 2006 17 4187 4199 10.1091/mbc.E06-01-0036 16855028 PMC1635340

[B47] Pacheco TR Coelho MB Desterro JMP Mollet I Carmo-Fonseca M In Vivo Requirement of the Small Subunit of U2AF for Recognition of a Weak 3' Splice Site Mol Cell Biol 2006 26 8183 8190 10.1128/MCB.00350-06 16940179 PMC1636752

[B48] Shav-Tal Y Zipori D PSF and p54(nrb)/NonO–multi-functional nuclear proteins FEBS Lett 2002 531 109 114 10.1016/S0014-5793(02)03447-6 12417296

[B49] Salton M Lerenthal Y Wang SY Chen DJ Shiloh Y Involvement of matrin 3 and SFPQ/NONO in the DNA damage response Cell Cycle 2010 9 1568 1576 10.4161/cc.9.8.11298 20421735

[B50] Li S Kuhne WW Kulharya A Hudson FZ Ha K Cao Z Dynan WS Involvement of p54(nrb), a PSF partner protein, in DNA double-strand break repair and radioresistance Nucleic Acids Res 2009 37 6746 6753 10.1093/nar/gkp741 19759212 PMC2777424

[B51] Bladen CL Udayakumar D Takeda Y Dynan WS Identification of the polypyrimidine tract binding protein-associated splicing factor.p54(nrb) complex as a candidate DNA double-strand break rejoining factor J Biol Chem 2005 280 5205 5210 15590677 10.1074/jbc.M412758200

[B52] Akhmedov AT Lopez BS Human 100-kDa homologous DNA-pairing protein is the splicing factor PSF and promotes DNA strand invasion Nucleic Acids Res 2000 28 3022 3030 10.1093/nar/28.16.3022 10931916 PMC108454

[B53] Cobbold LC Spriggs KA Haines SJ Dobbyn HC Hayes C de Moor CH Lilley KS Bushell M Willis AE Identification of internal ribosome entry segment (IRES)-trans-acting factors for the Myc family of IRESs Mol Cell Biol 2008 28 40 49 10.1128/MCB.01298-07 17967896 PMC2223313

[B54] Tiscornia G Mahadevan MS Myotonic dystrophy: the role of the CUG triplet repeats in splicing of a novel DMPK exon and altered cytoplasmic DMPK mRNA isoform ratios Mol Cell 2000 5 959 967 10.1016/S1097-2765(00)80261-0 10911990

[B55] Tsoi H Lau CK Lau KF Chan HY Perturbation of U2AF65/NXF1-mediated RNA nuclear export enhances RNA toxicity in polyQ diseases Hum Mol Genet 2011 20 3787 3797 10.1093/hmg/ddr297 21725067

[B56] Castelo-Branco P Furger A Wollerton M Smith C Moreira A Proudfoot N Polypyrimidine tract binding protein modulates efficiency of polyadenylation Mol Cell Biol 2004 24 4174 4183 10.1128/MCB.24.10.4174-4183.2004 15121839 PMC400487

[B57] Izquierdo JM Majos N Bonnal S Martinez C Castelo R Guigo R Bilbao D Valcarcel J Regulation of Fas alternative splicing by antagonistic effects of TIA-1 and PTB on exon definition Mol Cell 2005 19 475 484 10.1016/j.molcel.2005.06.015 16109372

[B58] Sharma S Falick AM Black DL Polypyrimidine tract binding protein blocks the 5' splice site-dependent assembly of U2AF and the prespliceosomal E complex Mol Cell 2005 19 485 496 10.1016/j.molcel.2005.07.014 16109373 PMC1635971

[B59] Sauliere J Sureau A Expert-Bezancon A Marie J The polypyrimidine tract binding protein (PTB) represses splicing of exon 6B from the beta-tropomyosin pre-mRNA by directly interfering with the binding of the U2AF65 subunit Mol Cell Biol 2006 26 8755 8769 10.1128/MCB.00893-06 16982681 PMC1636812

[B60] Marinescu V Loomis PA Ehmann S Beales M Potashkin JA Regulation of retention of FosB intron 4 by PTB PLoS One 2007 2 e828 10.1371/journal.pone.0000828 17786200 PMC1952174

[B61] Cosme RS Yamamura Y Tang Q Roles of polypyrimidine tract binding proteins in major immediate-early gene expression and viral replication of human cytomegalovirus J Virol 2009 83 2839 2850 10.1128/JVI.02407-08 19144709 PMC2655581

[B62] Robinson C Callow M Stevenson S Scott B Robinson BW Lake RA Serologic responses in patients with malignant mesothelioma: evidence for both public and private specificities Am J Respir Cell Mol Biol 2000 22 550 556 10783126 10.1165/ajrcmb.22.5.3930

[B63] Imai H Chan EK Kiyosawa K Fu XD Tan EM Novel nuclear autoantigen with splicing factor motifs identified with antibody from hepatocellular carcinoma J Clin Invest 1993 92 2419 2426 10.1172/JCI116848 8227358 PMC288425

[B64] Grosso AR Martins S Carmo-Fonseca M The emerging role of splicing factors in cancer EMBO Rep 2008 9 1087 1093 10.1038/embor.2008.189 18846105 PMC2581861

[B65] Korshunov A Remke M Gessi M Ryzhova M Hielscher T Witt H Tobias V Buccoliero AM Sardi I Gardiman MP Focal genomic amplification at 19q13.42 comprises a powerful diagnostic marker for embryonal tumors with ependymoblastic rosettes Acta Neuropathol 2010 120 253 260 10.1007/s00401-010-0688-8 20407781

[B66] Pfister S Remke M Castoldi M Bai AH Muckenthaler MU Kulozik A von Deimling A Pscherer A Lichter P Korshunov A Novel genomic amplification targeting the microRNA cluster at 19q13.42 in a pediatric embryonal tumor with abundant neuropil and true rosettes Acta Neuropathol 2009 117 457 464 10.1007/s00401-008-0467-y 19057917

[B67] Sayagues JM Abad MM Melchor HB Gutierrez ML Gonzalez-Gonzalez M Jensen E Bengoechea O Fonseca E Orfao A Munoz-Bellvis L Intratumoural cytogenetic heterogeneity of sporadic colorectal carcinomas suggests several pathways to liver metastasis J Pathol 2010 221 308 319 10.1002/path.2712 20527024

[B68] Kuuselo R Simon R Karhu R Tennstedt P Marx AH Izbicki JR Yekebas E Sauter G Kallioniemi A 19q13 amplification is associated with high grade and stage in pancreatic cancer Genes Chromosomes Cancer 2010 49 569 575 20232484 10.1002/gcc.20767 PMC2855495

[B69] Bayani J Marrano P Graham C Zheng Y Li L Katsaros D Lassus H Butzow R Squire JA Diamandis EP Genomic instability and copy-number heterogeneity of chromosome 19q, including the kallikrein locus, in ovarian carcinomas Mol Oncol 2011 5 48 60 10.1016/j.molonc.2010.08.002 20800559 PMC3110681

[B70] Bacolla A Wang G Jain A Chuzhanova NA Cer RZ Collins JR Cooper DN Bohr VA Vasquez KM Non-B DNA-forming sequences and WRN deficiency independently increase the frequency of base substitution in human cells J Biol Chem 2011 286 10017 10026 10.1074/jbc.M110.176636 21285356 PMC3060453

[B71] Thorsen K Mansilla F Schepeler T Oster B Rasmussen MH Dyrskjot L Karni R Akerman M Krainer AR Laurberg S Alternative splicing of SLC39A14 in colorectal cancer is regulated by the Wnt pathway Mol Cell Proteomics 2011 10 M110 002998 10.1074/mcp.M110.002998 PMC3013455 20938052

[B72] Goncalves V Matos P Jordan P The beta-catenin/TCF4 pathway modifies alternative splicing through modulation of SRp20 expression RNA 2008 14 2538 2549 10.1261/rna.1253408 18952824 PMC2590949

[B73] Shitashige M Naishiro Y Idogawa M Honda K Ono M Hirohashi S Yamada T Involvement of splicing factor-1 in beta-catenin/T-cell factor-4-mediated gene transactivation and pre-mRNA splicing Gastroenterology 2007 132 1039 1054 10.1053/j.gastro.2007.01.007 17383426

[B74] Sato S Idogawa M Honda K Fujii G Kawashima H Takekuma K Hoshika A Hirohashi S Yamada T beta-catenin interacts with the FUS proto-oncogene product and regulates pre-mRNA splicing Gastroenterology 2005 129 1225 1236 10.1053/j.gastro.2005.07.025 16230076

[B75] Lee HK Kwak HY Hur J Kim IA Yang JS Park MW Yu J Jeong S beta-catenin regulates multiple steps of RNA metabolism as revealed by the RNA aptamer in colon cancer cells Cancer Res 2007 67 9315 9321 10.1158/0008-5472.CAN-07-1128 17909039

[B76] Wagenaar RA Crawford HC Matrisian LM Stabilized beta-catenin immortalizes colonic epithelial cells Cancer Res 2001 61 2097 2104 11280772

