# Supplementary material for: Triplex DNA-binding proteins are associated with clinical outcomes revealed by proteomic measurements in patients with colorectal cancer
Source: Mol Cancer. 2012 Jun 8;11:38. doi: 10.1186/1476-4598-11-38 (PMC3537547; doi:10.1186/1476-4598-11-38)

Supplementary Figure 1.

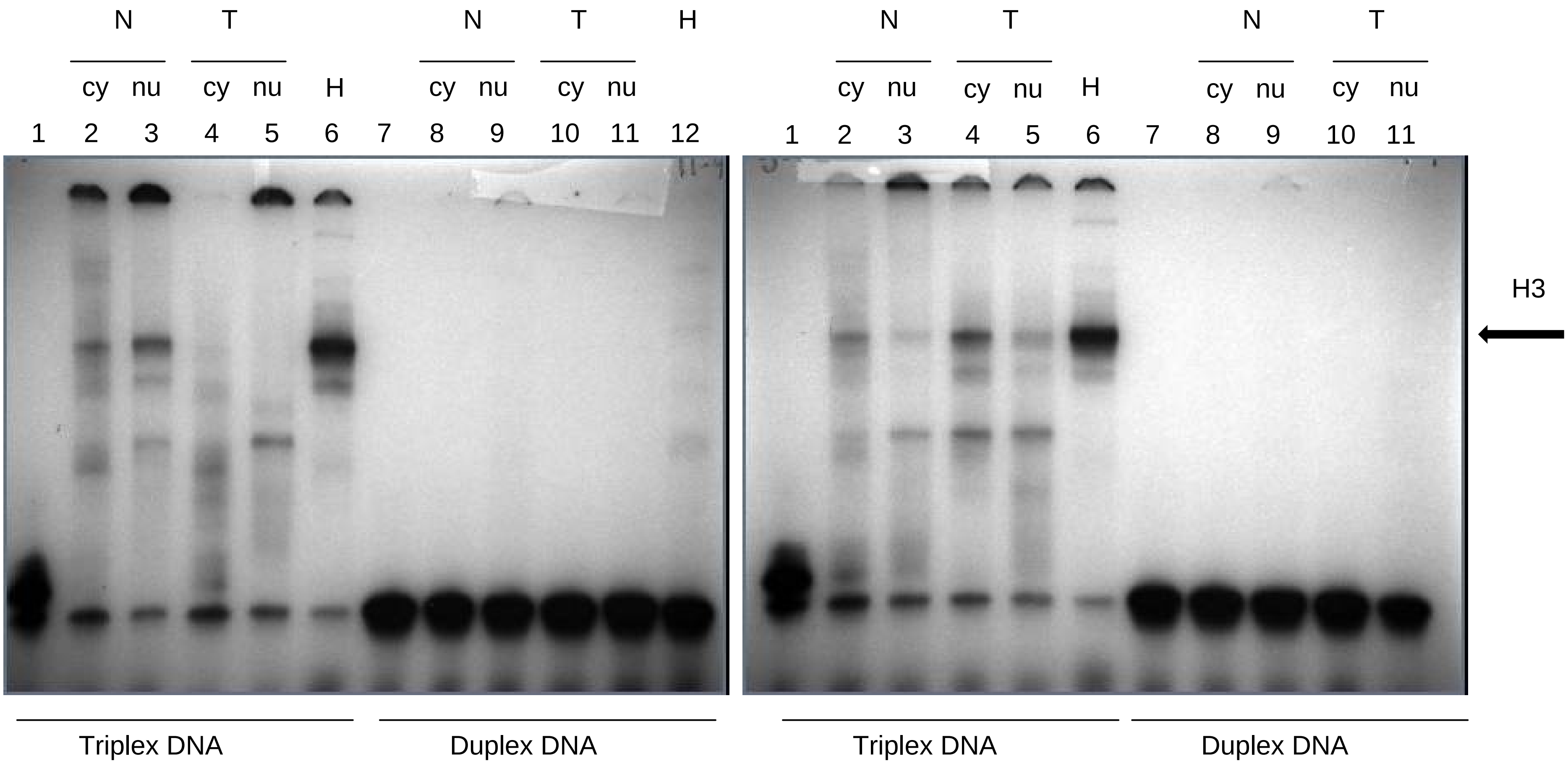

Supplementary Figure 2a

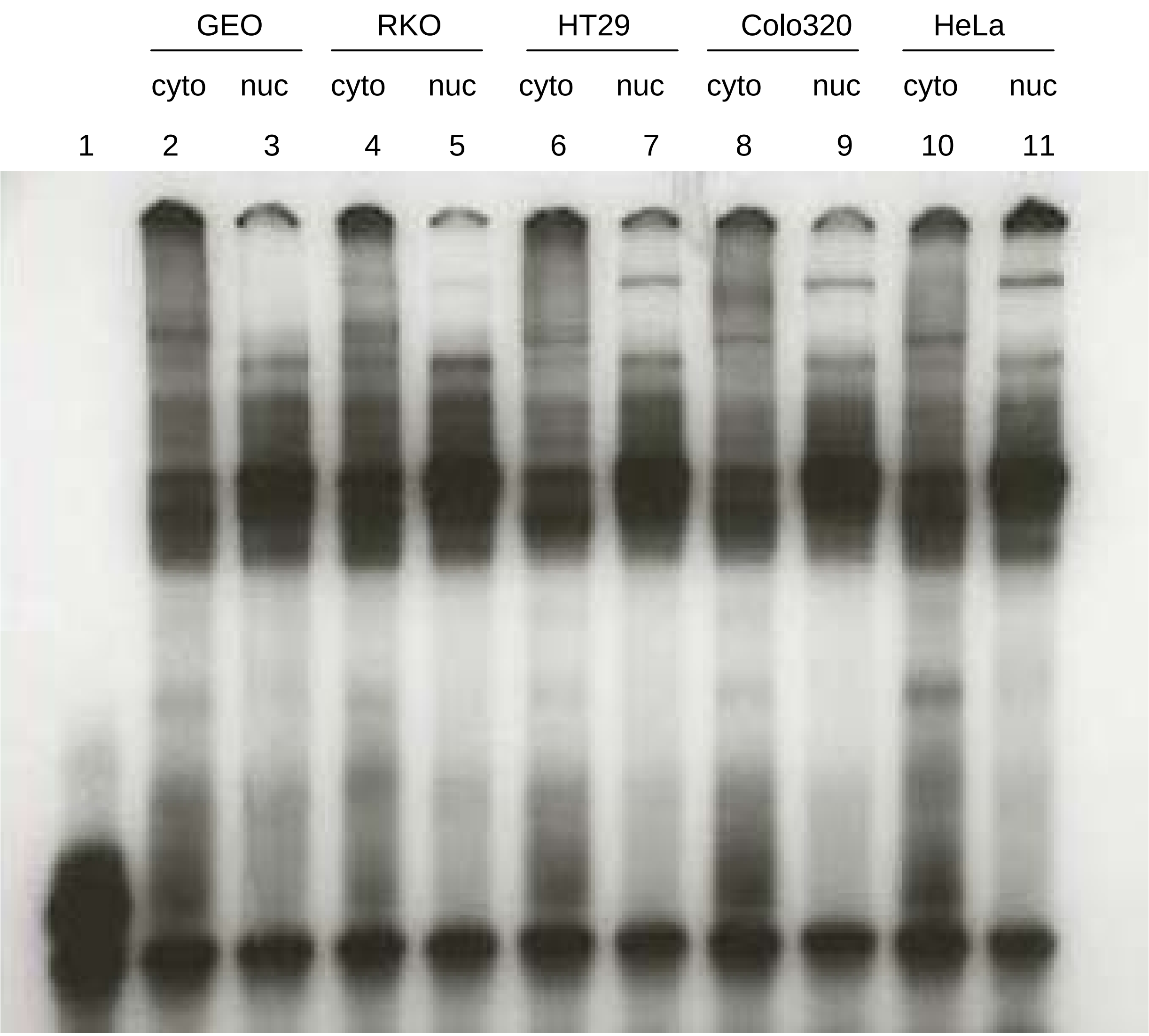

Supplementary Figure 2a continued

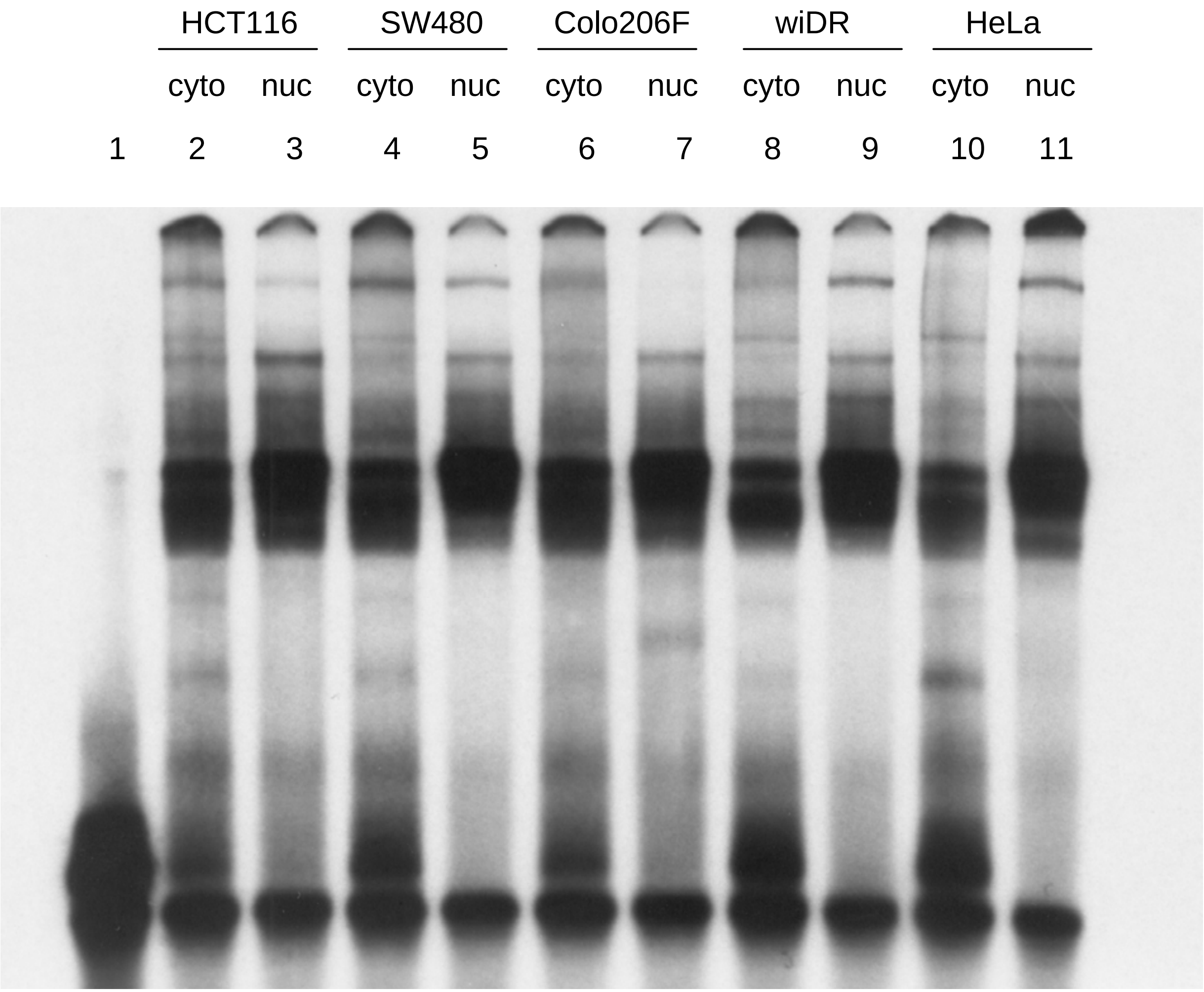

Supplementary Figure 2b

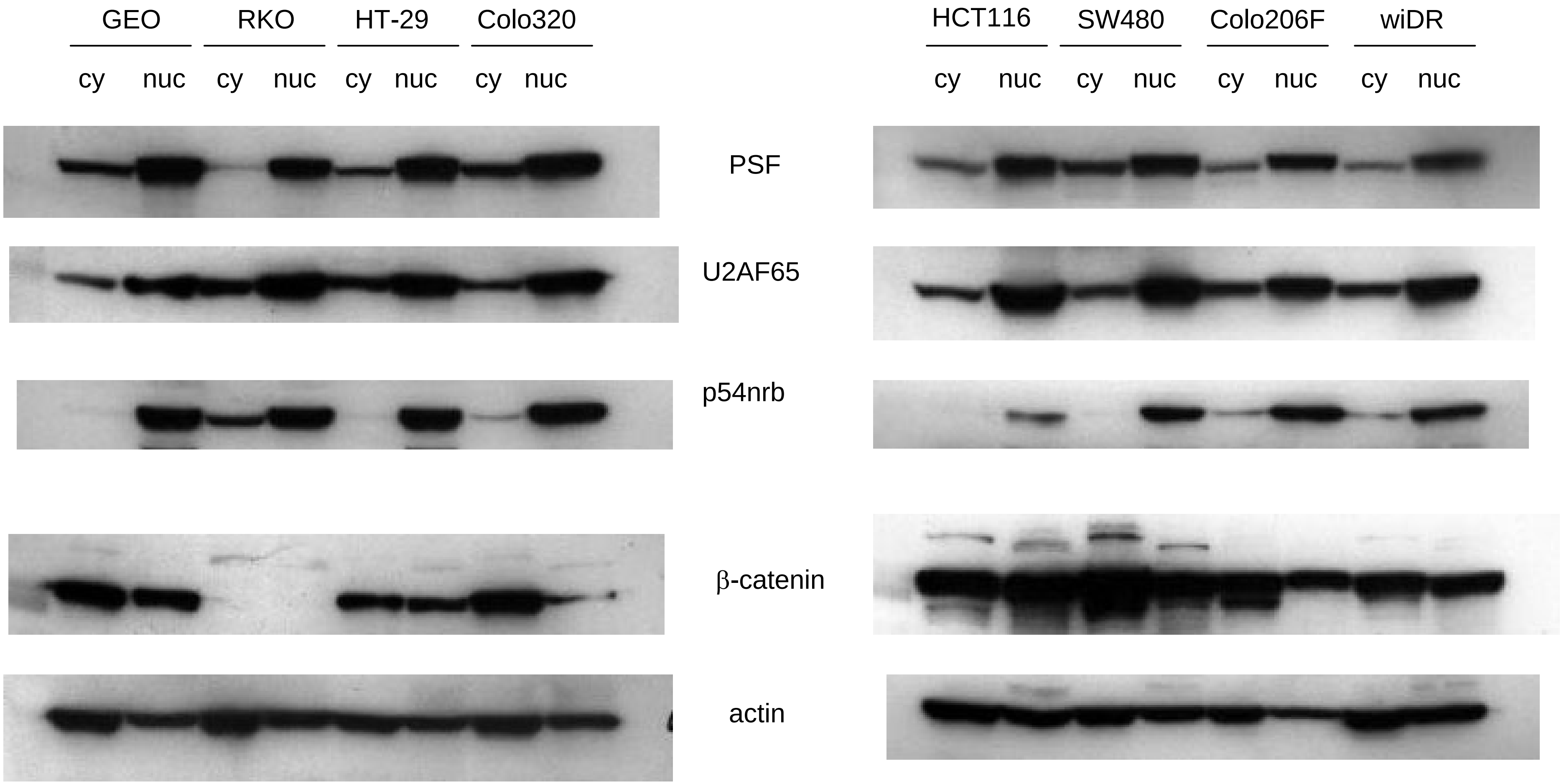

Supplementary Figure 3

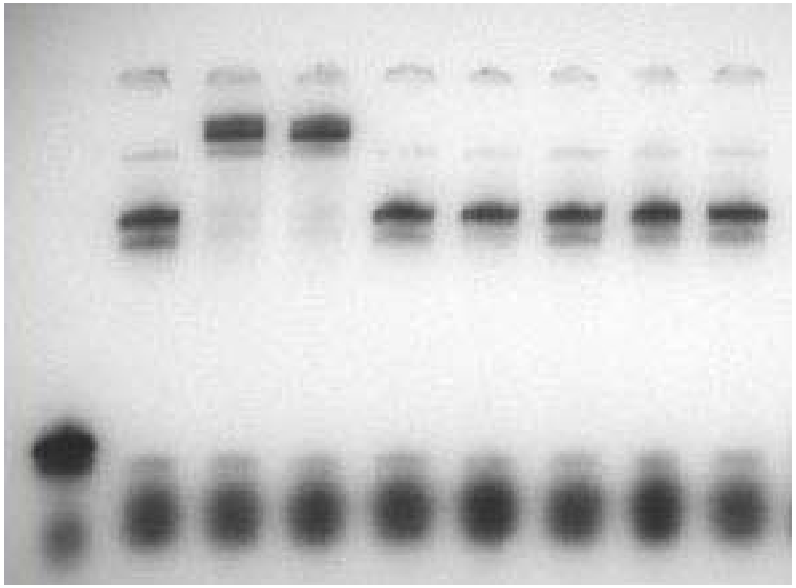

Supplementary Figure 4

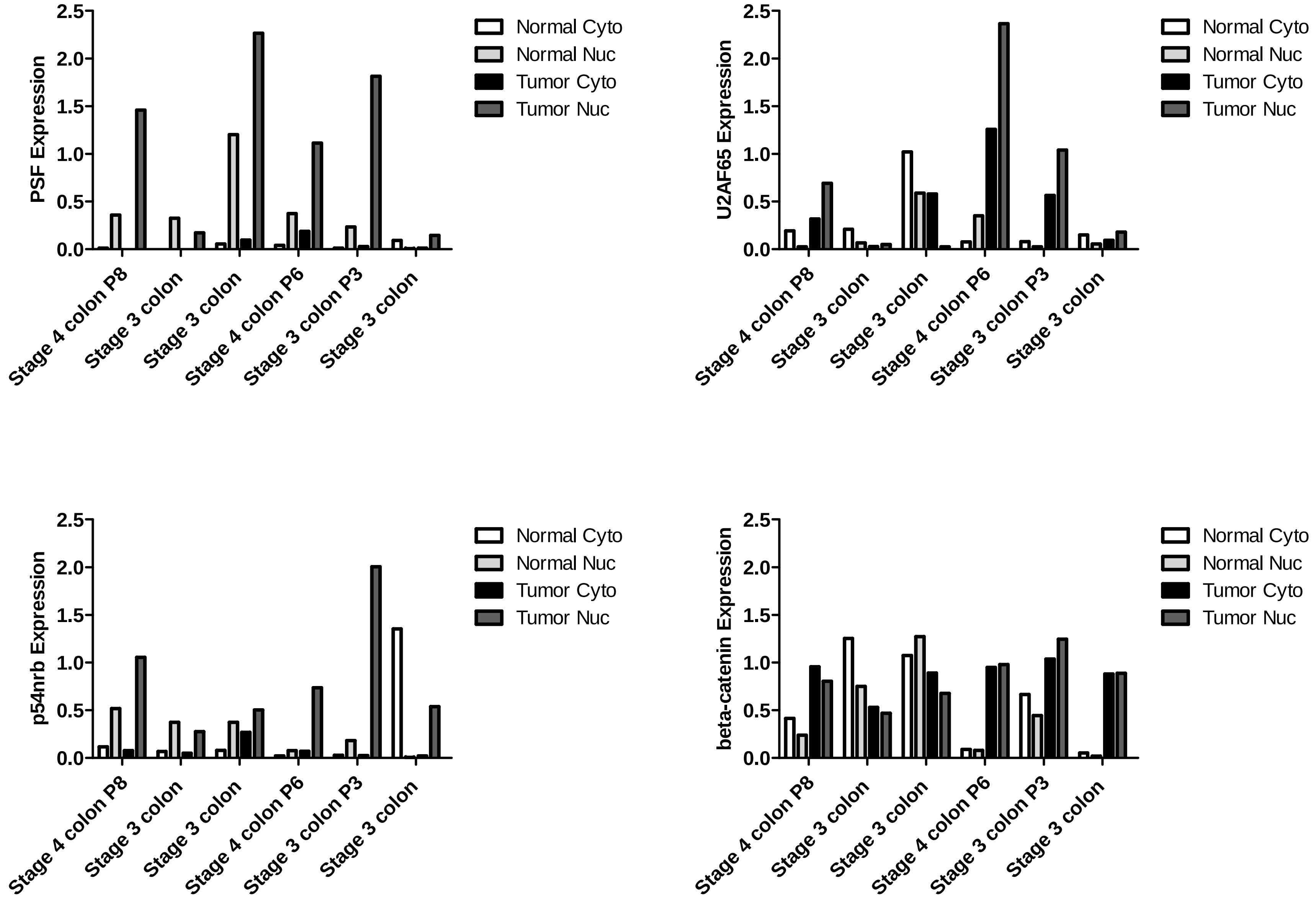

Supplementary Fig. 5

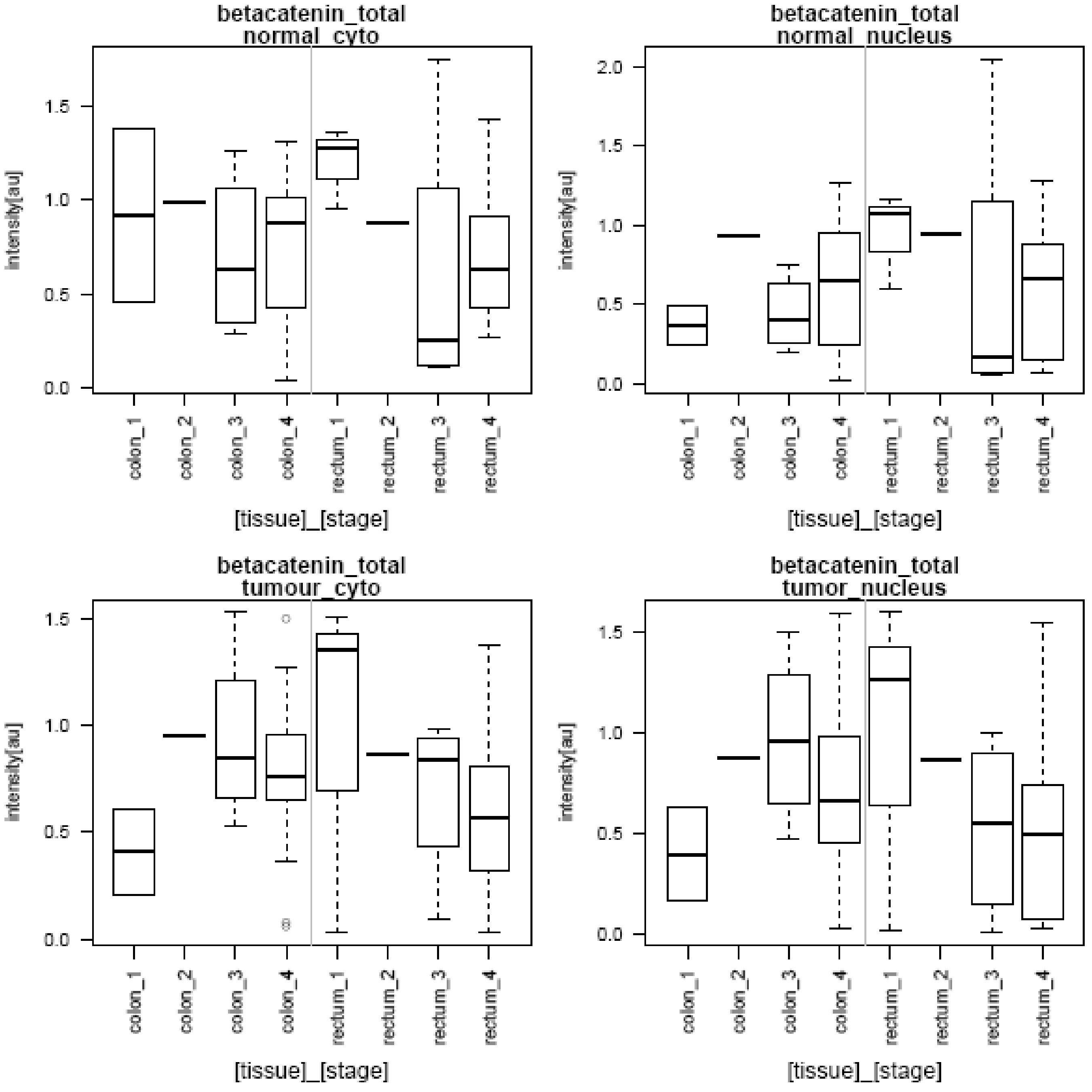

Supplement: Additional file 1 — Figure S1. Electrophoretic Mobility Shift Assay (EMSA) of patient tissue lysates and HeLa nuclear extract with triplex and parent duplex DNA probes. 33P‐labeled purine‐motif duplex or triplex DNA (1 nM) was complexed with 5 μg protein from normal tissue cytoplasmic (N cy), normal nuclear (N nu), tumor tissue cytoplasmic (T cy) or tumor nuclear (T nu) extracts of colorectal cancer patients. 1.25 μg HeLa nuclear extract (H) was used as a control in lanes 6 and 12. Purine triplex probe alone is in lane 1 and duplex probe alone is in lane 7. Figure S2a. Electrophoretic Mobility Shift Assay (EMSA) of Cytoplasmic and Nuclear Extracts from Eight Colorectal Cancer Cell Lines with Purine triplex DNA. 33P‐labeled purine‐motif triplex DNA (1 nM) was complexed with 1.25 μg total protein from cytoplasmic (cy) or nuclear (nuc) extracts from eight colorectal cancer cell lines. 1.25 μg HeLa cytoplasmic and nuclear extracts were used as positive (+) controls. Each reaction also contained 2 μg poly (dI‐dC) carrier DNA. The purine triplex DNA probe alone is shown in lane 1. Figure S2b. Western blots showing expression of three candidate triplex DNA‐binding proteins in eight colorectal cancer cell lines. Total protein (25 μg) from cytoplasmic (cy) and nuclear (nu) extracts from eight colorectal cancer cell lines were separated using 10% SDS‐PAGE and electro‐transferred to nitrocellulose membranes. Blots were incubated with the antibodies against PSF, U2AF65, p54nrb, beta‐catenin, and actin, then the appropriate secondary antibody and detected using chemiluminescence and autoradiography. Figure S3. Lack of a super‐shifted H3 band in RKO nuclear extract by super‐shift EMSA with antibodies against PSF and p54nrb. 33P‐labeled triplex DNA (1 nM) was complexed with 1.5 μg total protein from RKO nuclear extracts (lanes 2‐9). Lane 1, triplex DNA probe alone; Lane 2, no antibody; lane 3, 400 ng anti‐U2AF65 antibody MC3; lane 4, 1000 ng anti‐U2AF65 antibody MC3; lane 5, 400 ng anti‐PSF antibody; [file 1476-4598-11-38-S1.pdf]
