# Supplementary material for: Triplex DNA-binding proteins are associated with clinical outcomes revealed by proteomic measurements in patients with colorectal cancer
Source: Mol Cancer. 2012 Jun 8;11:38. doi: 10.1186/1476-4598-11-38 (PMC3537547; doi:10.1186/1476-4598-11-38)
Supplement: Additional file 2 — DB-Triplexdata. [file 1476-4598-11-38-S2.rtf]

I. Descriptive statistics

	Statistics

 	Cytoplasm Normal Tissue	Nucleus Normal Tissue	Total Normal Tissue	Cytoplasm Tumor	Nucleus Tumor	Total Tumor	Ratio Cytoplasm	Ratio Nucleus	Ratio Total	
N	Valid	64	64	64	64	64	64	63	63	63	
 	Missing	0	0	0	0	0	0	1	1	1	
Mean	,38605	,26402	,65006	,51188	,36055	,87247	18,32298	4,42233	2,03738	
Median	,38350	,22750	,59800	,50850	,36750	,83950	1,38800	1,39300	1,47200	
Std. Deviation	,208378	,178108	,364336	,292037	,234385	,477698	132,855710	17,087158	3,361328	
Range	1,074	,688	1,712	1,226	,923	1,902	1056,042	136,274	26,603	
Minimum	,000	,000	,000	,000	,000	,000	,003	,043	,026	
Maximum	1,074	,688	1,712	1,226	,923	1,902	1056,045	136,317	26,629	
In some cases mean was increased, when compared to median, that was due to some outliers.

Differences shown in the table were significant for all results:

	Ranks

 	N	Mean Rank	Sum of Ranks	
CytoplasmTumor - CytoplasmNormalTissue	Negative Ranks	24(a)	22,48	539,50	
 	Positive Ranks	39(b)	37,86	1476,50	
 	Ties	1(c)	 	 	
 	Total	64	 	 	
NucleusTumor - NucleusNormalTissue	Negative Ranks	23(d)	27,91	642,00	
 	Positive Ranks	40(e)	34,35	1374,00	
 	Ties	1(f)	 	 	
 	Total	64	 	 	
TotalTumor - TotalNormalTissue	Negative Ranks	22(g)	24,20	532,50	
 	Positive Ranks	41(h)	36,18	1483,50	
 	Ties	1(i)	 	 	
 	Total	64	 	 	
a  CytoplasmTumor < CytoplasmNormalTissue
b  CytoplasmTumor > CytoplasmNormalTissue
c  CytoplasmTumor = CytoplasmNormalTissue
d  NucleusTumor < NucleusNormalTissue
e  NucleusTumor > NucleusNormalTissue
f  NucleusTumor = NucleusNormalTissue
g  TotalTumor < TotalNormalTissue
h  TotalTumor > TotalNormalTissue
i  TotalTumor = TotalNormalTissue

	Test Statistics(b)

 	Cytoplasm Tumor vs. Cytoplasm Normal Tissue	Nucleus Tumor vs. Nucleus Normal Tissue	Total Tumor vs. Total Normal Tissue	
Z	-3,207(a)	-2,506(a)	-3,255(a)	
Asymp. Sig. (2-tailed)	,001	,012	,001	
a  Based on negative ranks.
b  Wilcoxon Signed Ranks Test

In all cases, tumours showed significantly higher binding activities.


II. Correlations of factors with each other (e.g. cytoplasmatic binding activity in tumor with cytoplasmatic binding activity in normal…)

Nonparametric Correlations


	Correlations

 	CytoplasmNormalTissue	NucleusNormalTissue	TotalNormalTissue	CytoplasmTumor	NucleusTumor	TotalTumor	
Spearman's rho	CytoplasmNormalTissue	Correlation Coefficient	1,000	,793(**)	,956(**)	,401(**)	,207	,347(**)	
 	 	Sig. (2-tailed)	.	,000	,000	,001	,101	,005	
 	 	N	64	64	64	64	64	64	
 	NucleusNormalTissue	Correlation Coefficient	,793(**)	1,000	,928(**)	,299(*)	,107	,231	
 	 	Sig. (2-tailed)	,000	.	,000	,016	,401	,066	
 	 	N	64	64	64	64	64	64	
 	TotalNormalTissue	Correlation Coefficient	,956(**)	,928(**)	1,000	,384(**)	,175	,318(*)	
 	 	Sig. (2-tailed)	,000	,000	.	,002	,166	,010	
 	 	N	64	64	64	64	64	64	
 	CytoplasmTumor	Correlation Coefficient	,401(**)	,299(*)	,384(**)	1,000	,645(**)	,914(**)	
 	 	Sig. (2-tailed)	,001	,016	,002	.	,000	,000	
 	 	N	64	64	64	64	64	64	
 	NucleusTumor	Correlation Coefficient	,207	,107	,175	,645(**)	1,000	,885(**)	
 	 	Sig. (2-tailed)	,101	,401	,166	,000	.	,000	
 	 	N	64	64	64	64	64	64	
 	TotalTumor	Correlation Coefficient	,347(**)	,231	,318(*)	,914(**)	,885(**)	1,000	
 	 	Sig. (2-tailed)	,005	,066	,010	,000	,000	.	
 	 	N	64	64	64	64	64	64	
**  Correlation is significant at the 0.01 level (2-tailed).
*  Correlation is significant at the 0.05 level (2-tailed).

As the table shows, many factors were correlated with each other.


III. Correlations of the factors with clinical features:

a)	UICC

	Correlations

 	UICC	RatioCytoplasm	RatioTotal	
Spearman's rho	UICC	Correlation Coefficient	1,000	,271(*)	,252(*)	
 	 	Sig. (2-tailed)	.	,032	,046	
 	 	N	64	63	63	
 	RatioCytoplasm	Correlation Coefficient	,271(*)	1,000	,894(**)	
 	 	Sig. (2-tailed)	,032	.	,000	
 	 	N	63	63	63	
 	RatioTotal	Correlation Coefficient	,252(*)	,894(**)	1,000	
 	 	Sig. (2-tailed)	,046	,000	.	
 	 	N	63	63	63	
*  Correlation is significant at the 0.05 level (2-tailed).
**  Correlation is significant at the 0.01 level (2-tailed).

UICC was only weakly (but significantly) associated with the cytoplasmatic ratio and the 
Total ratio (T/N), but not with the nuclear ratios or with any of the other results.

b)	T-Staging:

	Test Statistics(a)

 	CytoplasmTumor	NucleusTumor	TotalTumor	RatioCytoplasm	RatioNucleus	RatioTotal	
Mann-Whitney U	318,000	303,000	311,000	306,000	265,000	279,000	
Wilcoxon W	1644,000	1629,000	1637,000	1581,000	1540,000	1554,000	
Z	-,225	-,476	-,342	-,323	-1,019	-,781	
Asymp. Sig. (2-tailed)	,822	,634	,732	,747	,308	,435	
a  Grouping Variable: Tdicho

T-Staging was not associated with any of the factors (Asymptotic Significance is in all cases >0.05);

c)	N-Stage

	Test Statistics(a)

 	CytoplasmTumor	NucleusTumor	TotalTumor	RatioCytoplasm	RatioNucleus	RatioTotal	
Mann-Whitney U	369,500	414,500	389,000	322,000	313,000	310,500	
Wilcoxon W	1072,500	1117,500	1092,000	1025,000	1016,000	1013,500	
Z	-1,767	-1,156	-1,502	-2,220	-2,345	-2,380	
Asymp. Sig. (2-tailed)	,077	,248	,133	,026	,019	,017	
a  Grouping Variable: Ndicho

N-Stage was associated with ALL RATIOS; this means, all patients without lymph-node
Affection at diagnosis had significantly decreased binding ratios (T/N) in nucleus and in cytoplasm.

d)	M-Staging

	Test Statistics(a)

 	CytoplasmTumor	NucleusTumor	TotalTumor	RatioCytoplasm	RatioNucleus	RatioTotal	
Mann-Whitney U	278,000	247,000	257,000	237,000	276,000	253,000	
Wilcoxon W	1604,000	1573,000	1583,000	1512,000	1551,000	1528,000	
Z	-,893	-1,410	-1,243	-1,495	-,832	-1,223	
Asymp. Sig. (2-tailed)	,372	,159	,214	,135	,405	,221	
a  Grouping Variable: M

M-Staging at diagnosis was not significantly associated with any of the factors;


e)	Grading

	Test Statistics(a)

 	CytoplasmTumor	NucleusTumor	TotalTumor	RatioCytoplasm	RatioNucleus	RatioTotal	
Mann-Whitney U	183,500	183,000	179,000	229,000	218,000	221,000	
Wilcoxon W	1218,500	1218,000	1214,000	1264,000	1253,000	1256,000	
Z	-1,320	-1,330	-1,413	-,382	-,608	-,547	
Asymp. Sig. (2-tailed)	,187	,183	,158	,703	,543	,585	
a  Grouping Variable: G


Grading was not significantly associated with any of the results;


IV. Correlations with follow-up;

a)	Patients with distant metastases at any (!) time of the follow-up (this means patients
That had metastasis at diagnosis = M1 + patients with metastases during follow-up) 
Had significantly increased binding – activities:


	Test Statistics(a)

 	Cytoplasm Tumor	Nucleus Tumor	Total Tumor	Ratio Cytoplasm	Ratio Nucleus	Ratio Total	
Mann-Whitney U	342,500	309,500	321,000	323,000	357,000	332,000	
Wilcoxon W	1245,500	1212,500	1224,000	1184,000	1218,000	1193,000	
Z	-1,689	-2,156	-1,993	-1,845	-1,355	-1,716	
Asymp. Sig. (2-tailed)	,091	,031	,046	,065	,175	,086	
a  Grouping Variable: MetIrgendwann

Significant for nuclear binding activity (p=0.031) and for total binding activity (p=0.046);


b)	local or distant recurrence during follow-up (M1-Patients censored) was not significantly 
     associated with one of the results.


c)	Survival analyses (overall survival)


1: Overall survival with cut-off 1,393 (Ratio [Tumor (nucleus)] / Normal (nucleus)]), this means 
patients with ratio above 1,393 had significantly shorter survival:

	Overall Comparisons

 	Chi-Square	df	Sig.	
Log Rank (Mantel-Cox)	4,161	1	,041	
Breslow (Generalized Wilcoxon)	3,053	1	,081	
Test of equality of survival distributions for the different levels of RatNukDicMedian.

P=0.041; median follow-up: 28months.


                                            Follow-up (months)


I also tried other cut-off-values: the best one was 1.5; I think it we could use this cut-off in
The paper (it is just the median rounded up), the next survival curve is calculated with that 
Cut-off-value:


                                            Follow-up (months)

Significance: p=0.019;

	Overall Comparisons

 	Chi-Square	df	Sig.	
Log Rank (Mantel-Cox)	5,532	1	,019	
Breslow (Generalized Wilcoxon)	4,180	1	,041	
Test of equality of survival distributions for the different levels of RatioCUeinskommafünf.


Multivariate analyses were not significant in all cases calculated (but I think 
these data are really very good, with or without multivariante significance!!!)
 
