# Supplementary material for: Triplex DNA-binding proteins are associated with clinical outcomes revealed by proteomic measurements in patients with colorectal cancer
Source: Mol Cancer. 2012 Jun 8;11:38. doi: 10.1186/1476-4598-11-38 (PMC3537547; doi:10.1186/1476-4598-11-38)
Supplement: Additional file 3 — PK Statistical analysis Triplex. [file 1476-4598-11-38-S3.pdf]

Statistical analysis
This was performed with SPSS version 13.0 (SPSS Inc., Chicago, IL), two-sided, at significance level p=0.05; a statistical trend was defined as p≤0.1. The Mann-Whitney test was used to compare quantitative variables between two independent groups. Spearman correlations among continuous variables were computed. χ2-tests (Bonferroni-corrected) were applied for grouped/dichotomized variables. Survival was estimated by Kaplan-Meier analysis, and differences were tested by Mantel-Cox log-rank statistics; primary endpoints were *tumor-related death (disease- specific survival), death (overall survival),* tumor recurrence (recurrence-free survival, R0-patients only). The following variables were dichotomized: Protein level in nuclear and total extracts ratios as high levels in tumor vs. low level in tumor as compared to normal tissue, involved lymph nodes as pN0 vs. pN1-3, distant metastasis as M0 vs. M1, surgical curability as curative vs. non-curative resection (R0 vs. R1/2).

Results
Patient results.
65 patients (62 R0 resected; Table 1.) with primary colorectal cancer were analyzed for DNA Triplex binding proteins in primary tumors and adjacent normal mucosae. Median follow-up was 28,9 months (range, 0.1-67.9). Local tumor recurrence/metastasis was observed in 8 R0- resected patients; a total of 20 patients *died due to the tumor*.

Descriptive analysis for DNA Triplex binding proteins. Densimetric quantification of DNA Triplex binding proteins in cytoplasma and nuclear extracts of colorectal cancer tissue as described above:
Median DNA Triplex binding protein level in 65 patients normal mucosae was 0,37(ng/mg) protein (range, 0.01-1,074) for cytoplasma extracts and for nuclear extracts 0.23 ng/mg protein (range, 0.00-0,688, Fig.1A and Fig.1 B) The protein level of DNA Triplex binding protein in the same 65 patients´ tumor tissue revealed in cytoplasma lysatea a median of 0,51(ng/mg) protein (range, 0.00-1.226) and a median of 0.37 (range, 0.00- 0.923)in nuclear extracts(Fig.1 C and D).
DNA Triplex binding protein level in both compartments were significantly higher in tumors than in normal tissue (P < 0.001). The protein level of nuclear extracts highly correlates with the level measured in cytoplasmic lysates(P < 0.001). Addition of these two levels observed in the separated cell compartments created a representative score for each tissue sample (*Fig1. E and Fig 1.F*)
Elevated Protein level in tumors (range 1.01-26.68 fold in malignant tissue) was found in 64.1% (*Fig. 2A*) of patients(P=0.001, Wilcoxon rank). Additionally high levels in cytoplasma in tumor were strongly associated with low nuclear levels in adjacent normal tissue(P< 0.001, wilcoxon rank).

Tumor characteristics	*absolute (n=65)*	*relative(%)*
**Sex**		
male	48	73,8 %
female	17	26,2 %
**Localization**		
Colon	35	53,8 %
Rectum	30	46,2 %
**Resection**		
R0	62	95,4%
R1	3	4,6 %
**Grading**		
G1	2	3,1 %
G2	45	69,2 %
G3	11	16,9 %
Missing system	7	10,8 %
**Staging**		
pT1	3	4,6 %
pT2	10	15,4 %
pT3	38	58,5 %
pT4	14	21,5 %
**lymphnode affection**		
pN0		
pN1	38	58,5 %
pN2	17	26,2 %
	10	15,4 %
**Staging**		
M0	44	67,7%
M1	21	32,3%
**Status**		
alive	45	69,2%
dead	20	30,8%

Table 1. *Patient and tumor characteristics of the colorectal patients series(n=65)*

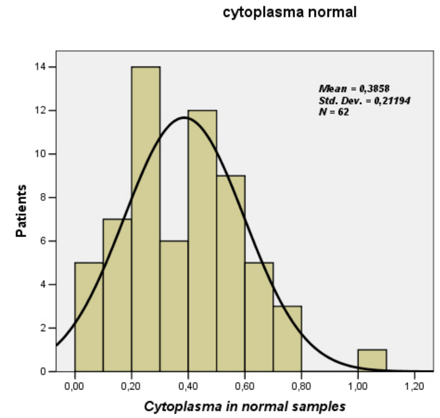

Fig.1A

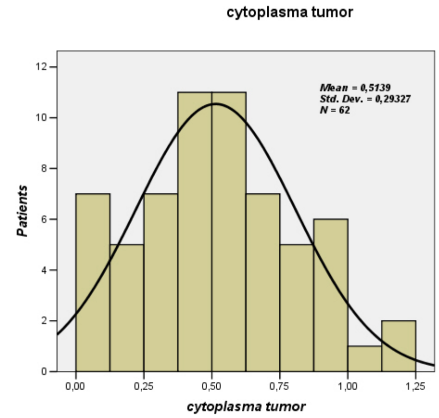

Fig.1D

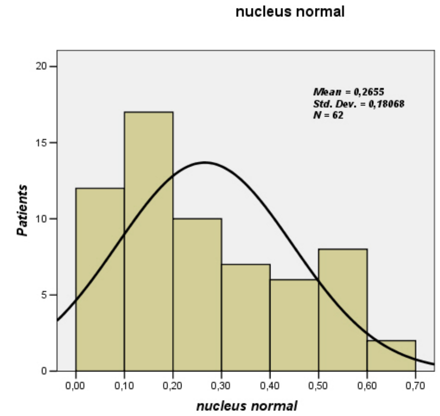

Fig.1B

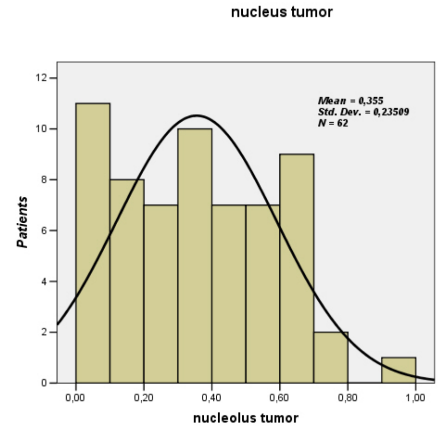

Fig.1E

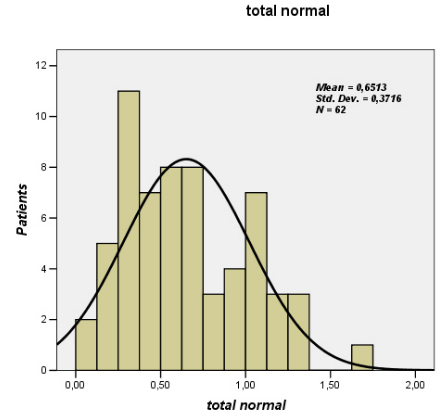

Fig.1C

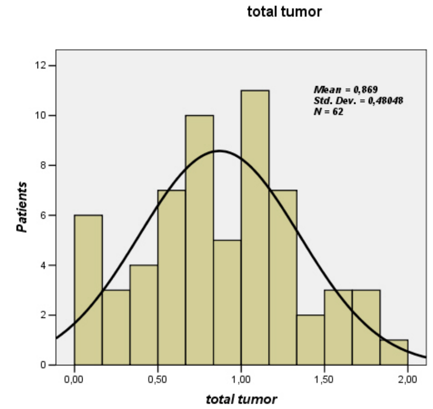

Fig.1F

Fig.1 *densimetric evaluation of DNA triplex binding protein of separate compartments in tumor and adjacent normal tissue: A) densimetric evaluation of cytoplasma in tumor adjacent normal sample:median 0,37(ng/mg) protein (range, 0.01-1,074) B) and for nuclear extracts: median 0.23(ng/mg) protein (range, 0.00-0,688) D) densimetric evaluation of cytoplasma in tumor: median 0.51(ng/mg) protein (range, 0.00-1.226) and E)nuclear extracts of tumor samples: median of 0.37 (ng/mg)(range, 0.00- 0.923), C)+F) densimetric evaluation of complete (cytoplasma+ nucleus) DNA triplex binding protein in normal and tumor tissue. Elevated Protein level in tumor as compared to normal tissue was observed in 64,1% patients (P=0.001, Wilcoxon rank). Additionally high levels in cytoplasma in tumor were strongly associated with low nuclear levels in adjacent normal tissue(P< 0.001, wilcoxon rank).*

Correlation with established tumorcharacteristics
To assess the association of the evaluated molecular parameter with established clinical tumor variables,$\chi 2$ analysis (Bonferroni corrected) was done with variables classified as described in Materials and Methods. Elevated DNA Triplex binding proteins in tumor as compared to associated normal sample correlated wirh Unio Internationalis Contra Cancrum (UICC) stages and with pN (P = 0.019, P = 0.037). Whereas no correlation was observed towards M-status. Exclusively in nuclear DNA Triplex binding protein level of tumor samples, the protein levels correlated highly significant with appearance of metastasis (P=0,008).
In univariate analysis the elevated presence of the DNA Triplex binding protein in both compartments (cyoplasma + nucleus) in tumor sample as compared to the associated normal sample showed a trend towards poorer overall survival (P<0,1). Whereas no association towards recurrence free survival in R0 resected cancer patients was observed (P=0.79).

Nevertheless, in Kaplan-Meier analysis (Mantel-Cox log-rank, median follow-up of 28.9 months), elevated triplex binding protein levels in nuclear lysates of tumor samples as compared to nuclear lysates of associated normal tissue corrleated with a shorter overall survival (P=0,045; Fig.3A). No association was found to recurrence free survival.
Interestingly, in Kaplan-Meier analysis, exclusively in tumor samples high protein levels in nuclear compartment was associated with poorer overall survival (mantel-Cox log-rank, P=0,036 Fig.3B)

Multivariate analysis DNA Triplex binding
multivariate analysis considering established clinical risk factors. Variables were included in the Cox proportional hazard model if they had shown univariate significance and compared to established risk factors such as pT, pN, pM status and the grading for cell differentiation. As key result, elevated DNA Triplex binding protein levels in nuclei of tumor cells as compared to nuclei of adjacent normal samples represent an independent prognostic factor for overall survival [P = 0.04; hazard ratio (HR), 3.474 95% confidenceinterval (95% CI), 1.06-11.37]. Surgical curability (curative versus non- curative resection) was excluded from the model due to skewness of distribution.

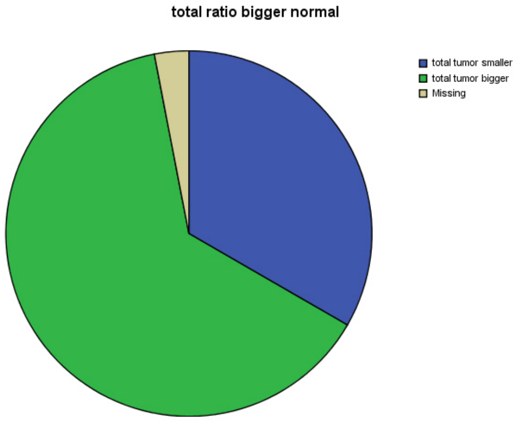

Fig.2A *distribution of DNA binding protein levels.In 64,1% of cases values for tumor exceed protein levels in normal tissue*

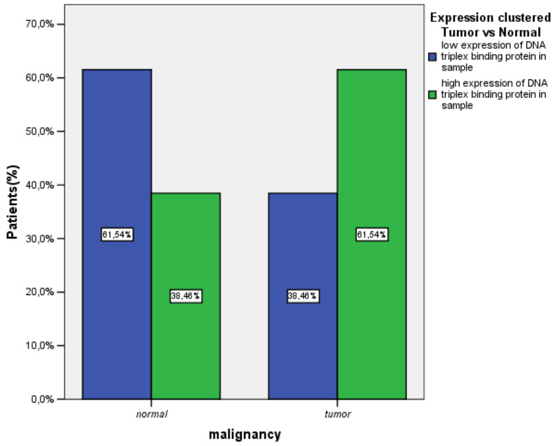

Fig.2B *bar chart, distribution of expression levels as compared to quality of tissue (tumor/normal), 61,54% of high expressing samples were tumor tissue. Whereas 61,54% of low expressing samples were normal*

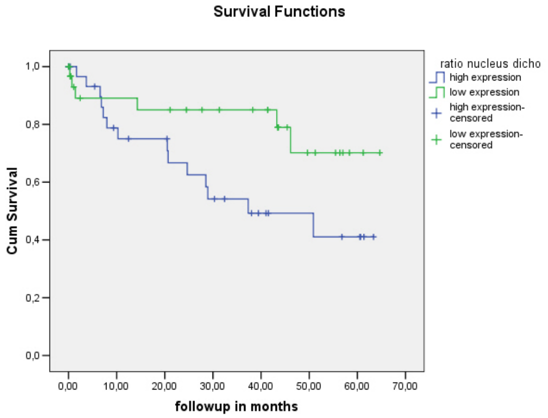

Fig.3A *expression of DNA Triplex binding protein in tumor nucleus as compared to normal nucleus, Mantel-Cox log rank P=0,045*

Fig.3B *expression in nucleus of tumor samples, Mantel-Cox log rank P=0,036*

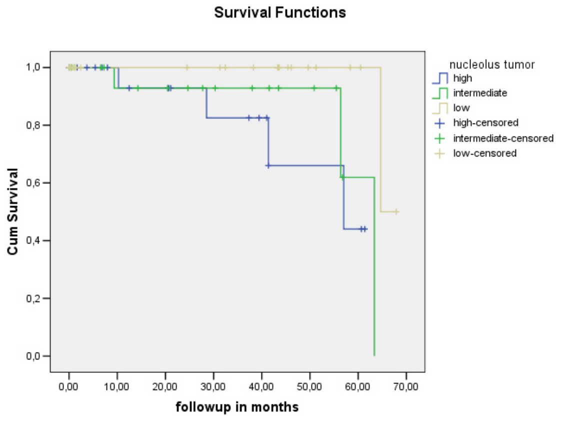

Table 2. *Cox Regression Multivariate analysis, comparing established prognostic parameters such as UICC TNM staging and differentiation. The resection status, curative vs. non-curative resection was not included in the model due to skewness of distribution,(R0, n=62, R1/2, n=3)*

	B	SE	Wald	df	Sig.	Exp(B)	95,0% CI for Exp(B)	
							Lower	Upper
pT: Tumorsize and state of infiltration	,633	,366	2,989	1	,084	1,883	,919	3,858
pN: affection of lymphnodes	-,306	,379	,651	1	,420	,736	,350	1,548
Differentiation G	,819	,575	2,034	1	,154	2,269	,736	6,997
Metastasis	,631	,580	1,182	1	,277	1,879	,603	5,862
Nuclear protein level in tumor as compared to normal nuclear level	1,245	,605	4,239	1	,040	3,474	1,062	11,371
