# Supplementary figures and images for: Triplex DNA-binding proteins are associated with clinical outcomes revealed by proteomic measurements in patients with colorectal cancer
Source: Mol Cancer. 2012 Jun 8;11:38. doi: 10.1186/1476-4598-11-38 (PMC3537547; doi:10.1186/1476-4598-11-38)

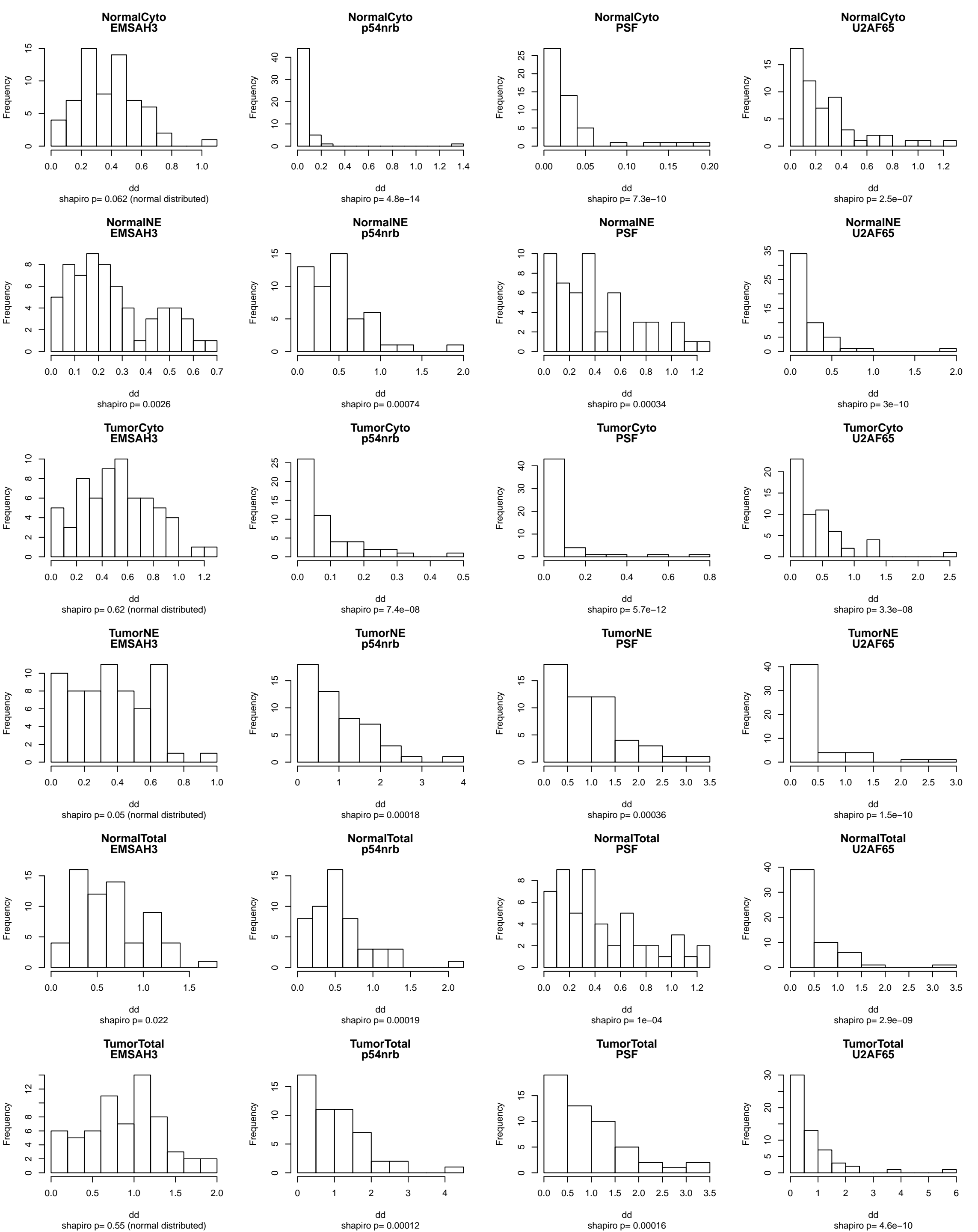

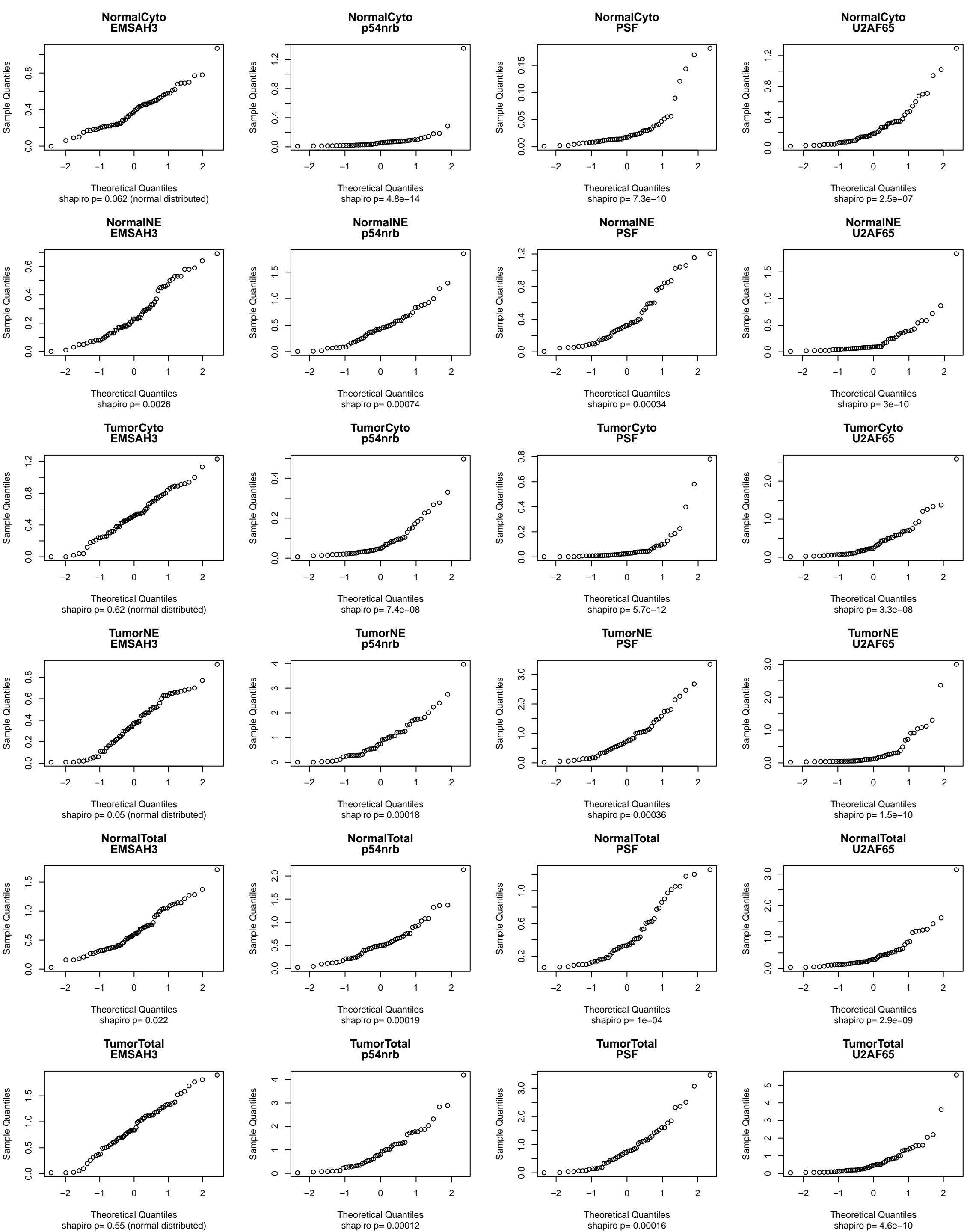

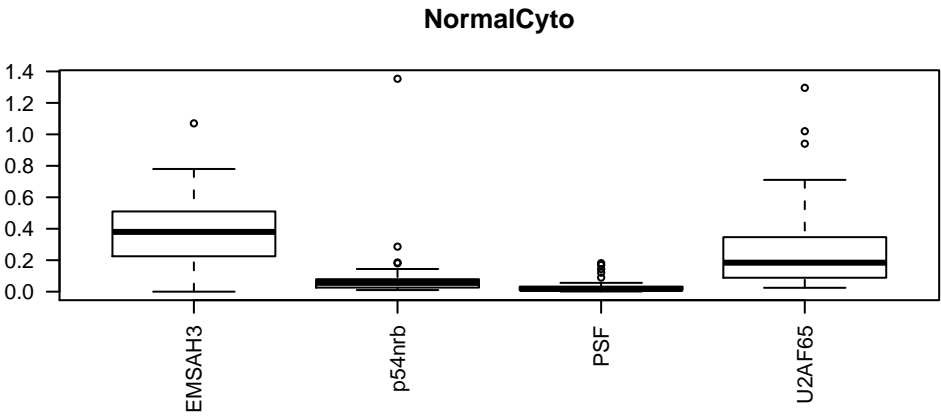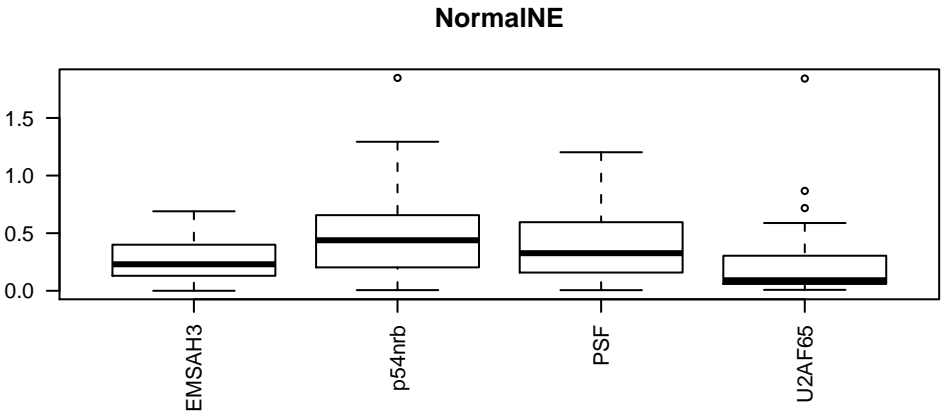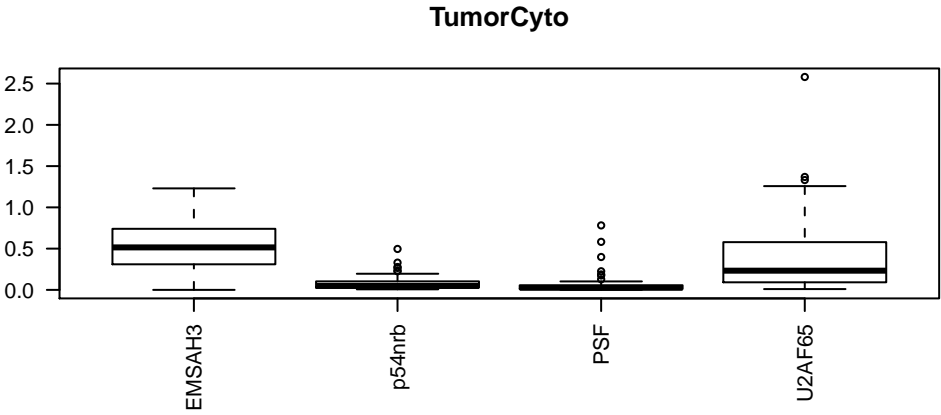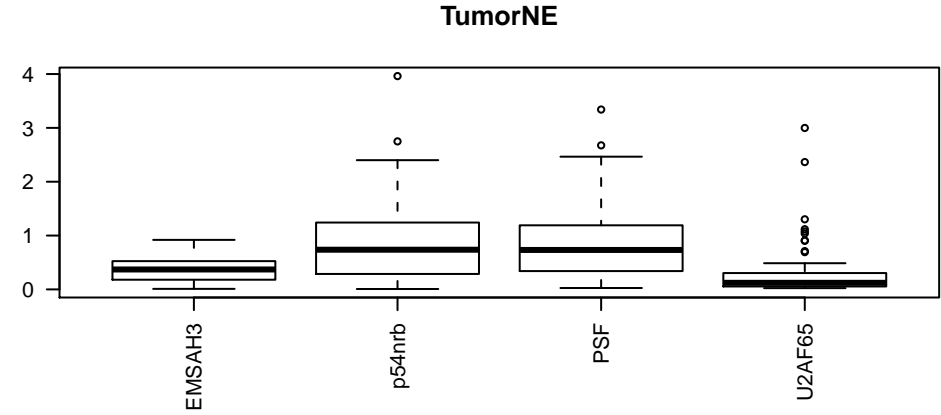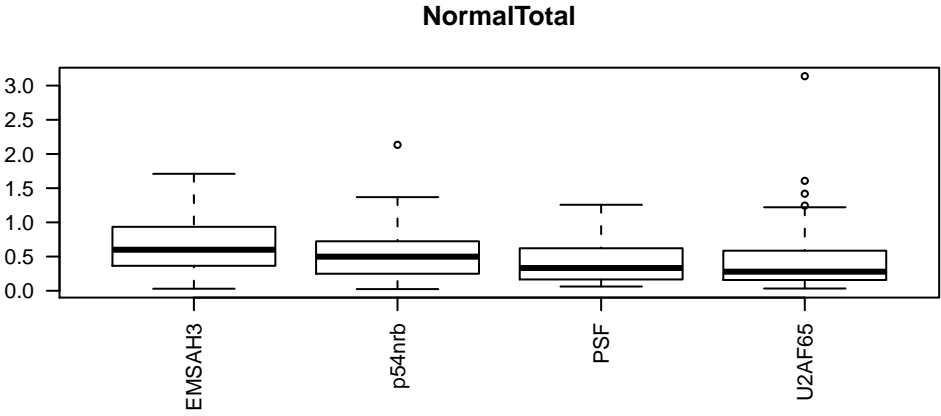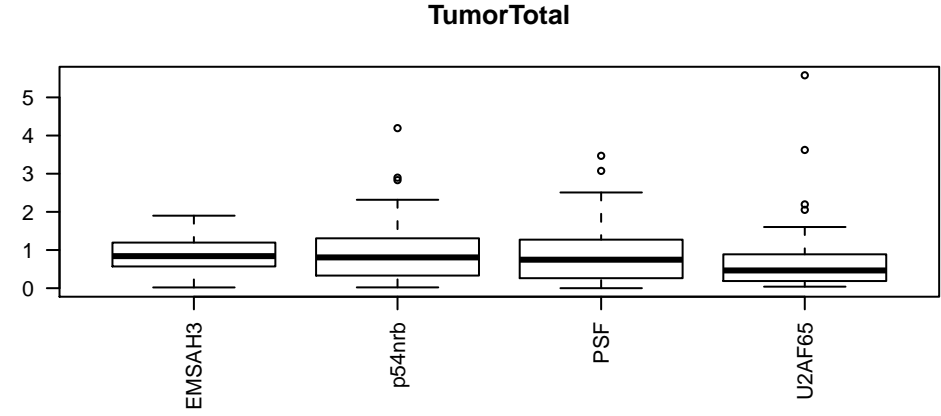

Supplement: Additional file 6 — histograms_proteins_groups. [file 1476-4598-11-38-S6.pdf]
