# Supplementary material for: Triplex DNA-binding proteins are associated with clinical outcomes revealed by proteomic measurements in patients with colorectal cancer
Source: Mol Cancer. 2012 Jun 8;11:38. doi: 10.1186/1476-4598-11-38 (PMC3537547; doi:10.1186/1476-4598-11-38)
Supplement: Additional file 7 — Table S1. RPPA antibodies and Spearman correlation p values. [file 1476-4598-11-38-S7.pdf]

Supplementary Table 1. RPPA antibodies and Spearman correlation p values

			EMSAH3	U2AF65	p 54nrb	PSF
**PKC alpha**	ab32376	abcam	**1**	**1**	**0.0466**	**0.1131**
**Ph-Erk**	4370	Cell Signaling	**0.468**	**0.00741**	**1**	**1**
**NF-κB p65**	sc-109	Santa Cruz	**5.46e-06**	**1.716e-05**	**1**	**1**
**Cyclin D1**	2922	Cell Signaling	**1**	**1**	**0.00021**	**8.19e-05**
**Ph Akt T308**	9275	Cell Signaling	**1**	**1**	**1**	**1**
**Ph Akt S473**	9271	Cell Signaling	**1**	**1**	**0.663**	**0.2457**
**Gsk3 beta**	9315	Cell Signaling	**4.66e-06**	**0.0179**	**1**	**1**
**c-Myc**	sc-764	Santa Cruz	**1**	**1**	**0.003**	**2.496e-06**
**JNK1**	610628	BD Biosciences	**1**	**1**	**0.00022**	**1.356e-06**
**PCNA**	sc-7907	Santa Cruz	**0.00024**	**7.02e-05**	**0.073**	**0.00466**
**Beta-catenin**	9562	Cell Signaling	**1.248e-11**	**8.97e-10**	**1**	**1**
**Ph Raf**	9421	Cell Signaling	**1**	**1**	**0.1633**	**0.00546**
**SRC**	2123	Cell Signaling	**0.00175**	**0.00358**	**1**	**1**
**p 38 alpha**	9212	Cell Signaling	**0.00207**	**0.00382**	**0.00055**	**1.94e-06**
**CDK4**	2341-1	Epitomics	**1**	**1**	**0.00468**	**0.00012**
**Akt1**	610860	BD Biosciences	**1**	**1**	**0.00105**	**5.85e-07**
**Akt2**	3063	Cell Signaling	**0.3042**	**1**	**0.0858**	**0.0507**
**ERK1**	AF1575	R&D Systems	**1**	**0.3549**	**1**	**1**
**ERK2**	sc-81458	Santa Cruz	**1**	**1**	**1**	**1**
**ErbB1**	2646	Cell Signaling	**1.443e-05**	**1.131e-07**	**0.00113**	**9.36e-06**
**Bcl-2**	ab32124	abcam	**1**	**0.00125**	**1**	**1**
**GRB2**	3972	Cell Signaling	**1**	**1**	**0.0238**	**0.00125**
**Her-2**	ab17	NeoMarkers	**0.624**	**0.0468**	**0.3588**	**0.01365**

**mTOR**	2983	Cell Signaling	**8.97e-05**	**0.000585**	**0.000858**	**7.02e-06**
**PARP**	AM-30	Calbiochem	**1**	**1**	**1**	**0.702**
**p 27**	610241	BD Biosciences	**0.1833**	**0.3003**	**1**	**1**
**P13K p110 alpha**	4249	Cell Signaling	**0.00585**	**0.000324**	**1**	**0.663**
**P13K p85 alpha**	ab40755	abcam	**1**	**1**	**1**	**1**
**P13K p110 beta**	ab32569	abcam	**1**	**1**	**0.0382**	**0.624**
**PLC gamma**	ab41433	abcam	**1**	**1**	**0.1131**	**9.75e-05**
**PP2A_A**	2039	Cell Signaling	**0.702**	**0.78**	**1**	**1**
**PP2A_B**	4953	Cell Signaling	**1**	**1**	**1**	**1**
**PTEN**	9552	Cell Signaling	**6.24e-10**	**3.354e-07**	**2.262e-06**	**1.092e-08**
**Rb**	9309	Cell Signaling	**1**	**0.2262**	**1**	**1**
**Smad23**	3102	Cell Signaling	**1**	**1**	**1**	**1**
**Stat3**	610189	BD Biosciences	**0.0226**	**0.663**	**0.00199**	**0.00012**
**Stat5**	sc-835	Santa Cruz	**5.46e-07**	**3.12e-05**	**0.00078**	**8.97e-06**
